# METTL3-dependent m^6^A modification of *GHR* mRNA regulates mitochondrial function through mitochondrial biogenesis during myoblast differentiation

**DOI:** 10.1016/j.psj.2025.105216

**Published:** 2025-04-25

**Authors:** Changbin Zhao, Bowen Hu, Zhijun Wang, Ze Zhang, Wen Luo, Hongmei Li, Xiquan Zhang

**Affiliations:** a State Key Laboratory for Conservation and Utilization of Subtropical Agro-Bioresources, Lingnan Guangdong Laboratory of Agriculture, College of Animal Science, South China Agricultural University, Guangzhou 510642, China; b National-Local Joint Engineering Research Center for Livestock Breeding, Guangdong Provincial Key Lab of Agro-Animal Genomics and Molecular Breeding, and Key Laboratory of Chicken Genetics, Breeding and Reproduction, Ministry of Agriculture, Guangzhou 510642, China

**Keywords:** *GHR*, *METTL3*, m^6^A, Mitochondrial biogenesis, Mitochondrial function

## Abstract

N6-methyl-adenosine (m^6^A) methylation has recently been shown to play a critical role in muscle development. We recently revealed that local *GHR* knockdown impairs mitochondrial function by inhibiting mitochondrial biogenesis, thereby repressing myoblast differentiation. And we identified m^6^A modification peaks in the *GHR* mRNA of chicken muscle tissue. However, whether m^6^A modification may regulate *GHR* mRNA expression to impinge on mitochondrial function through mitochondrial biogenesis during myoblast differentiation is lagging. We first predicted three potential m^6^A modification sites (*GHR*-139, *GHR*-203, *GHR*-385) on *GHR* mRNA through SRAMP online prediction website. We then confirmed that *GHR*-139 is the METTL3-dependent m^6^A modification site. Further, METTL3-dependent m^6^A modification down-regulated the *GHR* mRNA and protein expression, and blunted the *GHR* mediated GH-GHR-IGFs axis signal transduction during myoblast differentiation. We next revealed that METTL3-dependent m^6^A modification down-regulated *GHR* mRNA to inhibit mitochondrial biogenesis and impair mitochondrial function during myoblast differentiation. On the other hand, overexpression of *METTL3* alone also proved to inhibit the expression of *GHR* gene, while suppressing mitochondrial biogenesis and mitochondrial function. In terms of the m^6^A reader protein, we uncovered that m^6^A modification might regulate the *GHR* mRNA expression through three m^6^A reader proteins hnRNPR, hnRNPA3 and hnRNPM. In conclusion, our data corroborate that METTL3-dependent m^6^A modification down-regulates *GHR* mRNA expression to impair mitochondrial function by inhibiting mitochondrial biogenesis during myoblast differentiation.

## Introduction

As a crucial endocrine system regulating growth and development in the body, the growth hormone (GH)-growth hormone receptor (GHR)-insulin-like growth factor 1 (IGF1) axis plays a pivotal role in muscle development. GHR, a member of the type I cytokine receptor family, is widely expressed across various tissues and cell types (Hu et al., 2021). Previous studies have shown that GH binding to GHR activates the JAK2–STAT signaling pathway, leading to the transcription of specific genes such as *IGF1* and *IGF2*, thereby influencing cell proliferation and differentiation (Ranke and Wit, 2018). On the other hand, mitochondria mainly generate adenosine triphosphate (ATP) through oxidative phosphorylation (OXPHOS) to provide energy for myoblast differentiation (Rahman and Quadrilatero, 2021). We recently revealed that local *GHR* knockdown impairs mitochondrial function by inhibiting mitochondrial biogenesis via the IGF1-PI3K/AKT/CREB pathway, thereby repressing myoblast differentiation (Hu et al., 2023). Our data indicate that local *GHR* acts as a control value to maintain mitochondrial function by regulating mitochondrial biogenesis during myoblast differentiation.

N6-methyladenosine (m^6^A) is a prevalent post-transcriptional RNA modification in eukaryotic mRNA transcripts, such as mRNA splicing, export, translation, and degradation (Desrosiers et al., 1975; Jia et al., 2012; Jiang et al., 2021), which impinges on manifold biological processes including muscle development (Li et al., 2021). Dou et al. (2023) investigated m^6^A modifications in the longissimus dorsi muscle of pigs at different developmental stages and identified differential modifications in the periodic gene *CCND2* and the key transcription factor *MYOD1*, which are involved in muscle development and consequently influence muscle growth. Similarly, Feng et al. (2024) reported that METTL3-mediated m^6^A modification enhances *MYOD* expression, thereby promoting the activation of muscle satellite cells and skeletal muscle growth in adolescents. Moreover, m^6^A modification also influences skeletal muscle development by regulating the expression of non-coding RNAs (Mao et al., 2025; Su et al., 2025). It remains unclear whether the GH-GHR-IGF1 axis, a critical regulatory pathway for skeletal muscle development, is also affected by m^6^A modification.

The m^6^A modification requires well collaboration of methyltransferases (METTL3, METTL14 and METTL16), demethylases (FTO and ALKBH5) and methylated readers (YTHDF1/2/3, YTHDC1/2 and IGF2BP1/2/3) (Yue et al., 2015; Zaccara et al., 2019). METTL3 is the core catalytic subunit of the m^6^A methyltransferase complex that plays a pivotal catalytic role in m^6^A modification (Bokar et al., 1997). Research has found that METTL3 and IGFBP2-mediated m^6^A modifications of *NPR3* and *GHR* may play a profound regulatory role in rheumatoid arthritis (Xiao et al., 2022). However, the regulatory role in muscles remains unclear. Our previous data has revealed that there have m^6^A-modified peaks on the growth hormone receptor (*GHR*) mRNA through MeRIP-Seq in the pectoralis major muscle of Xinghua and White Recessive Rock chickens (Wang et al., 2024), suggesting that m^6^A modification might regulate *GHR* mRNA transcript during muscle development. Therefore, we wonder if m^6^A modification may regulate *GHR* mRNA expression to impinge on mitochondrial function through mitochondrial biogenesis during myoblast differentiation.

Based on this, we modulated the expression of the growth hormone receptor (*GHR*) gene and key methyltransferase genes in primary chicken myoblasts to investigate their roles in mitochondrial biogenesis and function during myoblast differentiation. Our results demonstrate that METTL3-dependent N⁶-methyladenosine (m⁶A) modification inhibits mitochondrial biogenesis by suppressing *GHR* mRNA expression during myoblast differentiation, ultimately leading to impaired mitochondrial function. Understanding the precise roles of m^6^A modification in regulating GH-GHR-IGFs growth axis to impinge on mitochondrial function during myoblast differentiation may provide new insights for the cultivated meat industry and potentially contribute to the improvement of muscle developmental disorders.

## Materials and methods

### Ethics statement

All animal experiments were conducted in accordance with the protocols approved by the Institutional Animal Care and Use Committee of South China Agricultural University (approval number: 2023c014; taroval date: Feb 18, 2023). All procedures complied with the regulations and guidelines set by the committee and were designed to minimize animal suffering.

### Cell culture

Chicken primary myoblasts (CPM) were isolated from the leg muscle of embryos on day 10 of incubation, as previously described (Luo et al., 2014). The CPM were cultured in growth medium (GM) consisting of RPMI-1640 medium (Gibco, USA), 15 % fetal bovine serum (Gibco, USA), and 0.2 % penicillin/streptomycin. When myoblasts reached 90 % confluence, the growth medium was replaced with differentiation medium (DM) including 2 % horse serum. All cells were maintained in a humidified incubator at 37°C with 5 % CO₂.

### RNA extraction and quantitative real-time PCR

Total RNA was extracted from cells using RNAiso reagent (Takara, Japan) according to the manufacturer’s instructions. RNA integrity was assessed by agarose gel electrophoresis, and concentration was measured using a Nanodrop 2000c spectrophotometer (Thermo, USA). Only RNA samples meeting quality requirements were used for further analysis. cDNA was synthesized from total RNA using the PrimeScript RT reagent Kit (Takara, Japan), following the manufacturer’s protocol. Quantitative real-time PCR (qRT-PCR) was performed using the MonAmp™ ChemoHS qPCR Mix (Monad, China) on a Bio-Rad CFX96 Real-Time PCR Detection System (Bio-Rad, USA), with β-actin as the internal reference gene. Primer sequences are listed in Table S1 and were synthesized by Sangon Biotech (Shanghai, China) Co., Ltd.

### DNA extraction and analysis of mtDNA copy number

Nuclear and mitochondrial DNA (mtDNA) were extracted from cells using the DNA Tissue Kit (Omega, USA), following the manufacturer’s instructions. DNA integrity was assessed via agarose gel electrophoresis, and concentration was determined using a Nanodrop 2000c spectrophotometer (Thermo, USA). Only DNA samples meeting quality criteria were used for subsequent analysis. qRT-PCR was performed using the MonAmp™ ChemoHS qPCR Mix (Monad, China) on a Bio-Rad CFX96 Real-Time PCR Detection System (Bio-Rad, USA), in accordance with the manufacturer's protocol. The relative mtDNA copy number was quantified by qRT-PCR, with each sample analyzed in duplicate. Mitochondrial genes *ND1* and *tRNA-Leu* were used as markers of total mitochondrial content, and the nuclear gene *β2M* served as the internal reference. Primer sequences are listed in Table S1 and were synthesized by Sangon Biotech (Shanghai, China) Co., Ltd.

### Plasmids construct

Overexpression vectors: To generate pcDNA3.1-*GHR*, pcDNA3.1-*METTL3*, pcDNA3.1-*METTL14* and pcDNA3.1-*METTL16*, cDNA encoding *GHR, METTL3, METTL14* and *METTL16* was amplified by PCR using chicken cDNA as the template, the forward primer contained an EcoRI site, and the reverse primer contained an XbaI site. The amplified fragment was purified, EcoRI/XbaI-digested, and ligated into EcoRI/XbaI sites in pcDNA3.1 vectors. Promoter reporter plasmids: pmir-GLO-*GHR*-139, pmirGLO-*GHR*-139-mut, pmirGLO-*GHR*-203, pmirGLO-*GHR*-203-mut were synthesized by a commercial company (Genecreate, China). The accuracy of all plasmid constructs was confirmed by Sanger DNA sequencing.

### Transfection

Cells were plated in culture plates (6 × 10^4^ cells per well of 12-well plate) and incubated overnight prior to the transfection experiment. Transfection was conducted once myoblasts reached 90 % cell confluence. To induce differentiation, the growth medium (GM) used for myoblast proliferation was replaced with differentiation medium (DM). Subsequently, the differentiating myoblasts were transfected using Lipofectamine 3000 reagent (Invitrogen, USA) according to the manufacturer’s instructions. All cells were subjected to further studies and analyses 48 h post-transfection.

### Western blot analysis

Cellular proteins were extracted by adding protease inhibitor (Beyotime, China) to protein lysis buffer (Beyotime, China) at a 1:100 ratio. The lysates were centrifuged at 12,000 × *g* for 5 minutes at 4 °C, and the resulting supernatant containing total cellular proteins was collected. Protein concentration was measured using the BCA Protein Assay Kit (Beyotime, China) according to the manufacturer’s instructions. The proteins were then separated on a 10 % SDS-PAGE and transferred onto a PVDF membrane. Subsequently, standard procedures were followed to probe the membrane with the respective antibodies. The Western blotting included the use of the following antibodies at the indicated dilutions: anti-GHR (bs-0654R; Bioss, China; 1:500), anti-PGC1α (bs-1832R; Bioss, China; 1:500), anti-NRF1 (12482-1-AP; Proteintech, USA; 1:500), anti-TOMM20 (AF1717; Beyotime, China; 1:500), anti-β-actin (bs-0061R; Bioss, China; 1:1000), goat anti-rabbit IgG-HRP (bs-0295 G; Bioss, China; 1:5000), and goat anti-mouse IgG-HRP (bs-0296G; Bioss, China; 1:5000).

### MeRIP sequencing and GHR m^6^A peak visualization

Three 7-week-old lean-type and obese-type broiler chickens were selected from each group, and their pectoral muscle tissue samples were collected. From each sample, 300 μg of RNA was extracted. After fragmentation, samples from each group underwent enrichment using an m^6^A antibody (Synaptic Systems, Göttingen, Germany) and non-enriched input processing. Subsequently, libraries were constructed for both the immunoprecipitated mRNA (IP group) and untreated mRNA (Input group) using the NEBNext UltraTM RNA Library Prep Kit (NEB, Ipswich, USA). Sequencing was performed on the Illumina HiSeq platform using standard protocols (paired-end reads, 150 bp in length). The raw sequencing data have been deposited in the CNCB (GSA database, accession number CRA008607). Based on the raw data, the m^6^A peak distribution of the *GHR* gene in both the IP and Input groups was visualized using Integrative Genomics Viewer (IGV, Broad Institute, Cambridge, USA).

### Methylated RNA immunoprecipitation assay

Methylated RNA immunoprecipitation (MeRIP) assay was performed as previously described (Li et al., 2022). In brief, RNA was first incubated with RNA fragmentation buffer in a thermal cycler at 70°C for 5 min. m^6^A antibody (CST, USA) was incubated with pre-washed protein A/G magnetic beads (MCE, China) overnight at 4°C. Second, the magnetic bead-antibody mixture was separated using a magnetic separation rack and washed twice with IP buffer. The mixture was resuspended in 500 μL of reaction solution and incubated at 4°C for 6-8 h. Third, the magnetic bead-antibody RNA mixture was washed twice in IP buffer, low-salt solution and high-salt solution at 4°C for 5 min each. The m^6^A-enriched RNA was eluted and purified using the RNeasy MinElute Cleanup Kit (Qiagen, Germany). Finally, the m^6^A-enriched RNA was validated through reverse transcription and PCR experiments, primers were shown in Table S2.

### RNA pull-down assay

Plasmids expressing full-length *GHR* mRNA containing F2 tagged and control vector were transfected into CPM. A F2 RNA-Protein Pull-Down Kit (FITGENE, China) was used in RNA-protein pull-down experiments according to the manufacturer’s instructions.

### Protein sample preparation and LC-MS/MS

Protein samples were initially treated for reduction by adding 4 µL of 0.05 M Tris(2-carboxyethyl) phosphine (Sigma, USA) and incubating at 60°C for 1 h. Subsequently, alkylation was achieved by adding 2 µL of 55 mM S-methyl methanethiosulfonate (MMTS, Sigma) followed by incubation in darkness at room temperature for 45 minutes. Sample cleanup and buffer exchange were performed using 10 kDa cut-off ultrafiltration tubes. After initial centrifugation at 12,000 g for 20 minutes, the samples were washed twice with 100 µL of UA buffer (8 M urea, pH 8.5, Sigma) and then three times with 100 µL of 0.25 M Triethylammonium bicarbonate (TEAB, Sigma), with each wash step followed by centrifugation at 12,000 g for 20 minutes. For enzymatic digestion, 50 µL of 0.5 M TEAB containing trypsin (Promega, USA) was added at an enzyme-to-protein mass ratio of 1:50. This mixture was incubated overnight (12 h) at 37°C. The following day, digestion was continued by adding trypsin at a 1:100 mass ratio and incubating for an additional 4 h at 37°C. The resulting peptide mixture was transferred to a fresh collection tube, and the filtrate containing the peptides was collected via centrifugation. The collected peptides were then dried using low-temperature vacuum evaporation.

Dried peptide fractions were reconstituted in loading buffer (0.1 % formic acid (Sigma), 2 % acetonitrile (Fisher, USA)) for liquid chromatography-tandem mass spectrometry (LC-MS/MS) analysis. Peptide separation was carried out on a Dionex Ultimate 3000 RSLCnano system (Fisher) equipped with a C18 reversed-phase column (150 mm length, 75 µm inner diameter, 3 µm resin). A binary solvent system consisting of mobile phase A (0.1 % formic acid in water) and mobile phase B (0.1 % formic acid in 80 % ACN) was used. Peptides were eluted over 65 minutes using a linear gradient from 5 % to 50 % mobile phase B at a flow rate of 300 nL/min. The LC eluate was directly analyzed using a Q Exactive Hybrid Quadrupole-Orbitrap mass spectrometer (Fisher) operating in information-dependent acquisition (IDA) mode. Full MS scans (MS1) were acquired over the m/z range of 350-1,800 at a resolution of 70,000, with a maximum ion accumulation time of 250 ms. From each MS1 scan, up to 20 of the most intense precursor ions were selected for fragmentation via higher-energy collisional dissociation (HCD) using a normalized collision energy setting of 30. Tandem mass spectra (MS/MS) were acquired at a resolution greater than 17,500, with a minimum precursor accumulation time of 100 ms and dynamic exclusion enabled for 20 seconds. The raw data have been deposited in the CNCB and are accessible through OMIX series accession number OMIX009631.

### Protein identification and bioinformatics analysis

The raw mass spectrometry data were acquired in RAW format and converted into MGF format using Proteome Discoverer 1.4 (version 1.4.0.288, Fisher). The MGF file and the protein database (https://www.uniprot.org/taxonomy/9031) were input into ProteinPilot™ Software 4.5 (version 1656, SCIEX, USA) for mass spectrometry identification. After subtracting the control group's data from the identified protein data of the experimental group, the remaining differential proteins were considered the target binding proteins. Gene Ontology (GO, http://geneontology.org) and Kyoto Encyclopedia of Genes and Genomes (KEGG, http://www.kegg.jp) were used to annotate all identified proteins. GO and KEGG enrichment analyses were conducted using Fisher's exact test, with FDR correction applied for multiple comparisons. Enriched GO terms and KEGG pathways were considered statistically significant at *p* < 0.05.

### Mito-tracker green staining and hoechst 33342 staining

Mitochondria were stained using Mito-Tracker Green (MTG) (Beyotime, China), and nuclei were counterstained with Hoechst 33342 (Beyotime, China). At 48 h post-transfection, cells were rinsed twice with PBS and incubated with MTG for 30 minutes. After two additional PBS washes, cells were incubated with Hoechst 33342 for 10 minutes, followed by two final rinses with PBS. Fluorescence images were captured from five randomly selected fields per sample using a TE2000-U fluorescence microscope (Nikon, Japan), and image analysis was performed using NIS-Elements software.

### Detection of reactive oxygen species

Intracellular reactive oxygen species (ROS) levels were measured using the ROS Assay Kit (Beyotime, China) following the manufacturer’s instructions. The fluorescence intensity of dichlorofluorescin (DCF) was quantified using a multifunctional microplate reader (BioTek, USA) to evaluate ROS production in myoblasts.

### Detection of ATP content

ATP levels in myoblasts were measured using an ATP Detection Kit (Beyotime, China) following the manufacturer’s instructions. The luminescence intensity was detected using a multifunctional microplate reader (BioTek, USA) to quantify intracellular ATP content.

### Detection of mitochondrial membrane potential

Mitochondrial membrane potential (ΔΨm) in myoblasts was assessed using the JC-1 Assay Kit (Beyotime, China), following the manufacturer’s instructions. For each sample, five randomly selected fields were imaged using a TE2000-U fluorescence microscope (Nikon, Japan). The captured images were subsequently analyzed using NIS-Elements software.

### Statistical analysis

All experiments were performed in at least three independent replicates. Graphical representations were generated using GraphPad Prism v9.0 (GraphPad Software, USA). Data are presented as mean ± standard error of the mean (SEM). Statistical analyses were conducted using Student’s t-test. A *p*-value of < 0.05 was considered statistically significant. Significance levels are indicated as follows: *p < 0.05, **p < 0.01, ***p < 0.001, and ns indicates no significance.

## Results

### Verification of potential methyltransferase and m^6^A modification sites on GHR mRNA in chicken primary myoblasts (CPM)

**Fig. 1:**
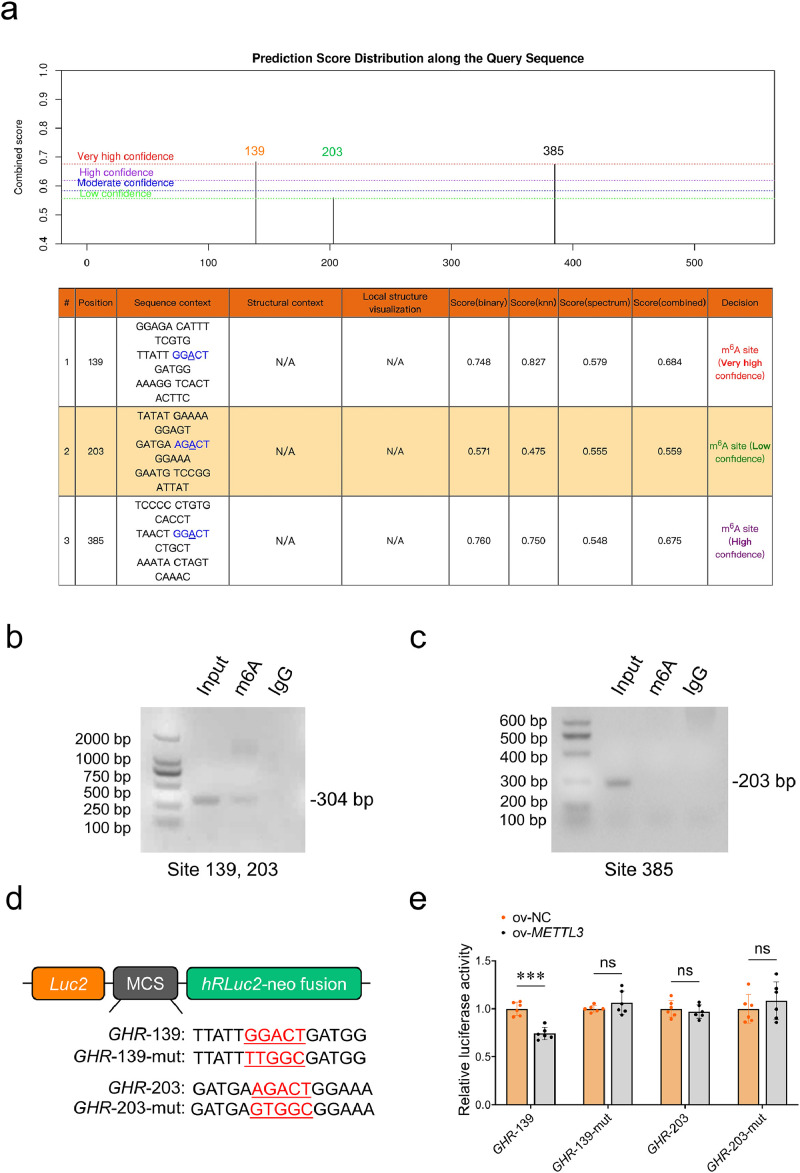
Verification of potential methyltransferase and m^6^A modification sites on *GHR* mRNA in CPM. (a) Prediction of potential m^6^A modification sites on *GHR* mRNA through online prediction SRAMP prediction server (http://www.cuilab.cn/sramp). (b) MeRIP-PCR assays were applied to assess the m^6^A methylation modification sites on *GHR*-139 and *GHR*-203 after *METTL3* overexpression during myoblast differentiation. (c) MeRIP-PCR assays were applied to assess the m^6^A methylation modification sites on *GHR*-385 after *METTL3* overexpression during myoblast differentiation. (d, e) Relative luciferase activities of CPM co-transfected with plasmids containing wild-type or mutant *GHR* mRNA and *METTL3* cDNA. Firefly luciferase activities were measured and normalized to renilla luciferase activity (*n* = 6). Data are shown as mean ± SEM, ***p < 0.001, ns means no significant.

In previous studies, we identified m^6^A modification peaks on *GHR* mRNA in the pectoralis muscle of chickens through MeRIP-Seq (Fig. S1a), suggesting that m^6^A modification of *GHR* mRNA may play a significant role in muscle development. We first explored potential m^6^A modification sites on *GHR* mRNA by the online prediction SRAMP prediction server (http://www.cuilab.cn/sramp). Three potential m^6^A modification sites, *GHR*-139, *GHR*-203, and *GHR*-385, were predicted on *GHR* mRNA (Fig. 1**a**). To confirm the locations of m^6^A modification sites, *METTL3* was overexpressed in differentiating myoblasts, with high overexpression efficiency verified (Fig. S1b), followed by MeRIP-PCR to validate the predicted sites. Gel electrophoresis revealed that, following m^6^A immunoprecipitation (IP), the PCR-amplified fragment containing the *GHR*-139 and *GHR*-203 sites exhibited a distinct 304 bp band (Fig. 1**b**), indicating specific enrichment of this region and the presence of m^6^A modifications. In contrast, no corresponding band was observed for the amplified fragment containing *GHR*-385 after m^6^A IP compared to the input group (Fig. 1**c**), suggesting that this region was not enriched and did not contain m^6^A modification sites. To further investigate the m^6^A modification enzymes of *GHR* mRNA and their specific modification sites, we constructed four pmirGLO vectors containing either the wild-type or mutated versions of *GHR*-139 and *GHR*-203, and co-transfected them with *METTL3, METTL14*, and *METTL16* in CPM (Fig. 1**d**). The dual fluorescence assay revealed that *METTL3* overexpression significantly inhibited the expression of the wild-type *GHR*-139 reporter gene but had no effect on the mutant *GHR*-139 reporter gene. *METTL3* overexpression did not affect the expression of either the wild-type or mutant *GHR*-203 (Fig. 1**e**). Moreover, overexpression of *METTL14* and *METTL16* had no impact on either the wild-type or mutant forms of *GHR*-139 and *GHR*-203 (Fig. S1c, d). These results indicate that the regulation of *GHR* expression levels might rely on the METTL3-dependent m^6^A modification at the *GHR*-139 site during myoblast differentiation.

### METTL3-dependent m^6^A modification regulates GHR mRNA and protein expression, and the mRNA expression involved in GH-GHR-IGFs axis during CPM differentiation

**Fig. 2:**
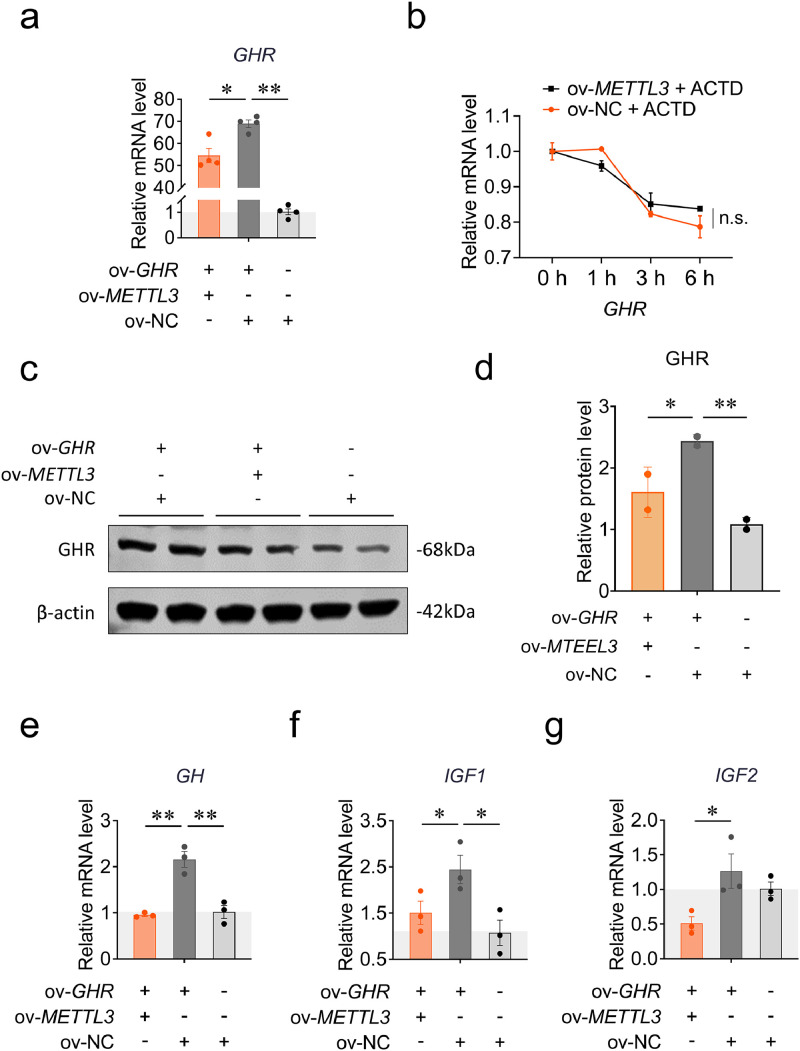
METTL3-dependent m^6^A modification regulates *GHR* mRNA and protein expression, and the mRNA expression involved in the GH-GHR-IGFs axis during CPM differentiation. (a) The *GHR* mRNA expression was measured by RT-qPCR at 48 h after co-transfection with pcDNA3.1-*METTL3*, pcDNA3.1-*GHR* and pcDNA3.1 (*n* = 4). (b) The RNA stability of *GHR* mRNA was detected by RT-qPCR with *METTL3* overexpression treated with ACTD during myoblast differentiation (*n* = 3). (c, d) The GHR protein expression was measured by western blots at 48 h after co-transfection with pcDNA3.1-*METTL3*, pcDNA3.1-*GHR* and pcDNA3.1 (*n* = 2). (e) The *GH* mRNA expression was measured by RT-qPCR at 48 h after co-transfection with pcDNA3.1-*METTL3*, pcDNA3.1-*GHR* and pcDNA3.1 (*n* = 3). (f) The *IGF1* mRNA expression was measured by RT-qPCR at 48 h after co-transfection with pcDNA3.1-*METTL3*, pcDNA3.1-*GHR* and pcDNA3.1 (*n* = 3). (g) The *IGF2* mRNA expression was measured by RT-qPCR at 48 h after co-transfection with pcDNA3.1-*METTL3*, pcDNA3.1-*GHR* and pcDNA3.1 (*n* = 3); Data are shown as mean ± SEM, *p < 0.05, **p < 0.01.

To further investigate the METTL3-dependent m^6^A modification roles in the regulation of *GHR* mRNA, we co-transfected pcDNA3.1-*METTL3* and pcDNA3.1-*GHR* during CPM differentiation. The *GHR* mRNA and protein expression were all decreased in the co-transfection group compared with the *GHR* overexpression group (Fig. 2**a, c, d**). In addition, we successfully overexpressed the *METTL3* gene in CPM (Fig. S2a), and found that this significantly inhibited *GHR* expression (Fig. S2b). We then determined the stability of *GHR* mRNA by actinomycin D (ACTD). The *GHR* mRNA expression did not change significantly in the co-transfection group compared with the *GHR* overexpression group (Fig. 2**b**), indicating that METTL3-dependent m^6^A modification did not influence the stability of *GHR* mRNA during myoblast differentiation. On the other hand, we determined the mRNA expression involved in the GH-GHR-IGFs axis by RT-qPCR. The *GH, IGF1* and *IGF2* expression were all significantly down-regulated in the co-transfection group compared with the *GHR* overexpression group (Fig. 2**e-f**). Taken together, these results indicate that METTL3-dependent m^6^A modification suppresses the *GHR* mRNA and protein expression, and blunts the GHR-mediated GH-GHR-IGFs signaling during myoblast differentiation.

### METTL3-dependent m^6^A modification of GHR mRNA regulates the mRNA and protein expression involved in mitochondrial biogenesis during CPM differentiation

**Fig. 3:**
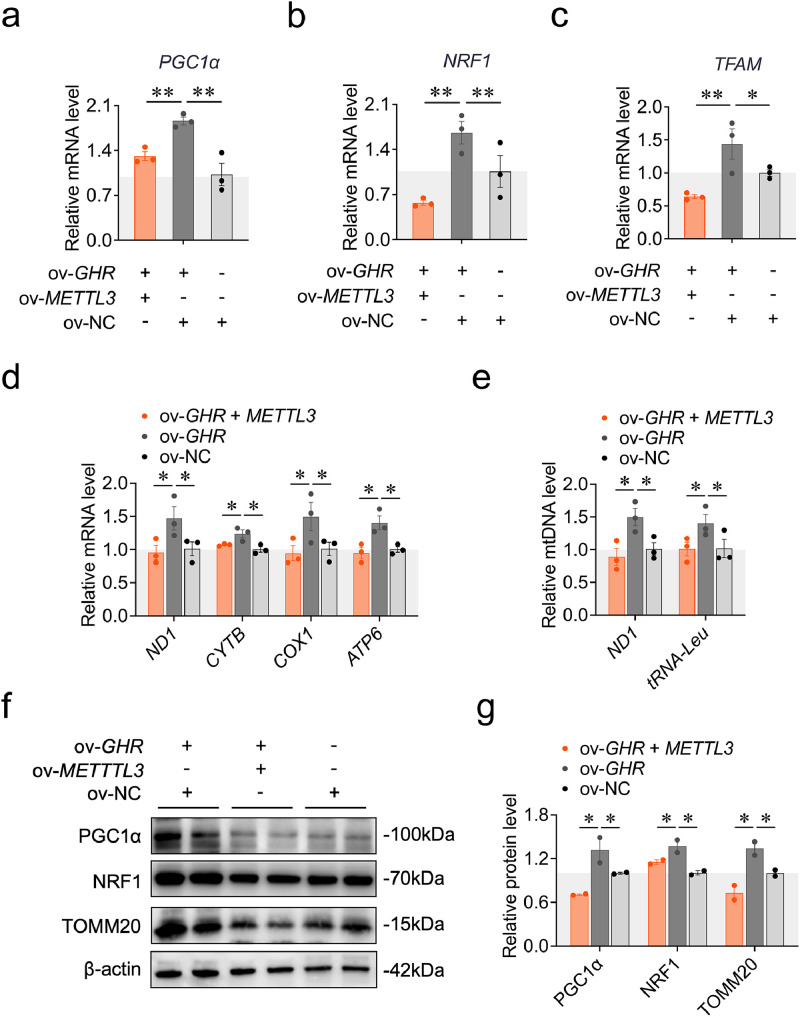
METTL3-dependent m^6^A modification of *GHR* mRNA regulates the mRNA and protein expression involved in mitochondrial biogenesis during CPM differentiation. (a) The *PGC1α* mRNA expression was measured by RT-qPCR at 48 h after co-transfection with pcDNA3.1-*METTL3*, pcDNA3.1-*GHR* and pcDNA3.1 (*n* = 3). (b) The *NRF1* mRNA expression was measured by RT-qPCR at 48 h after co-transfection with pcDNA3.1-*METTL3*, pcDNA3.1-*GHR* and pcDNA3.1 (*n* = 3). (c) The *TFAM* mRNA expression was measured by RT-qPCR at 48 h after co-transfection with pcDNA3.1-*METTL3*, pcDNA3.1-*GHR* and pcDNA3.1 (*n* = 3). (d) The mtDNA transcription was measured by RT-qPCR at 48 h after co-transfection with pcDNA3.1-*METTL3*, pcDNA3.1-*GHR* and pcDNA3.1 (*n* = 3). (e) The mtDNA copy number was measured by qPCR at 48 h after co-transfection with pcDNA3.1-*METTL3*, pcDNA3.1-*GHR* and pcDNA3.1 (*n* = 3). (f, g) The PGC1α, NRF1, TOMM20 protein expression was measured by western blots at 48 h after co-transfection with pcDNA3.1-*METTL3*, pcDNA3.1-*GHR* and pcDNA3.1 (*n* = 2). Data are shown as mean ± SEM, *p < 0.05, **p < 0.01.

We asked whether METTL3-dependent m^6^A modification of *GHR* mRNA regulates mitochondrial biogenesis during myoblast differentiation. We first examined the expression of mitochondrial biogenesis markers, including *PGC1α, NRF1*, and *TFAM*, following co-transfection in CPM. The *PGC1α, NRF1* and *TFAM* expression were significantly down-regulated in the co-transfection group compared with the *GHR* overexpression group (Fig. 3**a-c**). Consistently, single *METTL3* overexpression significantly inhibited *PGC1α, NRF1* and *TFAM* expression (Fig. S2e). We then investigated the effects of METTL3-dependent m^6^A modification of *GHR* mRNA on mtDNA transcription and replication. The expression levels of mtDNA-encoded genes (*ND1, CYTB, COX1, ATP6*) and mtDNA copy number (indicated by *ND1* and *tRNA-Leu*) were significantly decreased in the co-transfection group compared to the *GHR* overexpression group (Fig. 3**d, e**). Similar results were observed upon *METTL3* overexpression alone (Fig. S2c, d). Finally, we assessed the protein levels of mitochondrial biogenesis marker genes. The PGC1α, NRF1 and TOMM20 protein levels were reduced in the co-transfection group compared with the *GHR* overexpression group (Fig. 3**f, g**), similarly to the results observed with *METTL3* overexpression (Fig. S2f, g). Collectively, these findings suggest that METTL3-mediated m⁶A modification of *GHR* mRNA suppresses mitochondrial biogenesis during myoblast differentiation.

### METTL3-dependent m^6^A modification of GHR mRNA regulates mitochondrial function during CPM differentiation

**Fig. 4:**
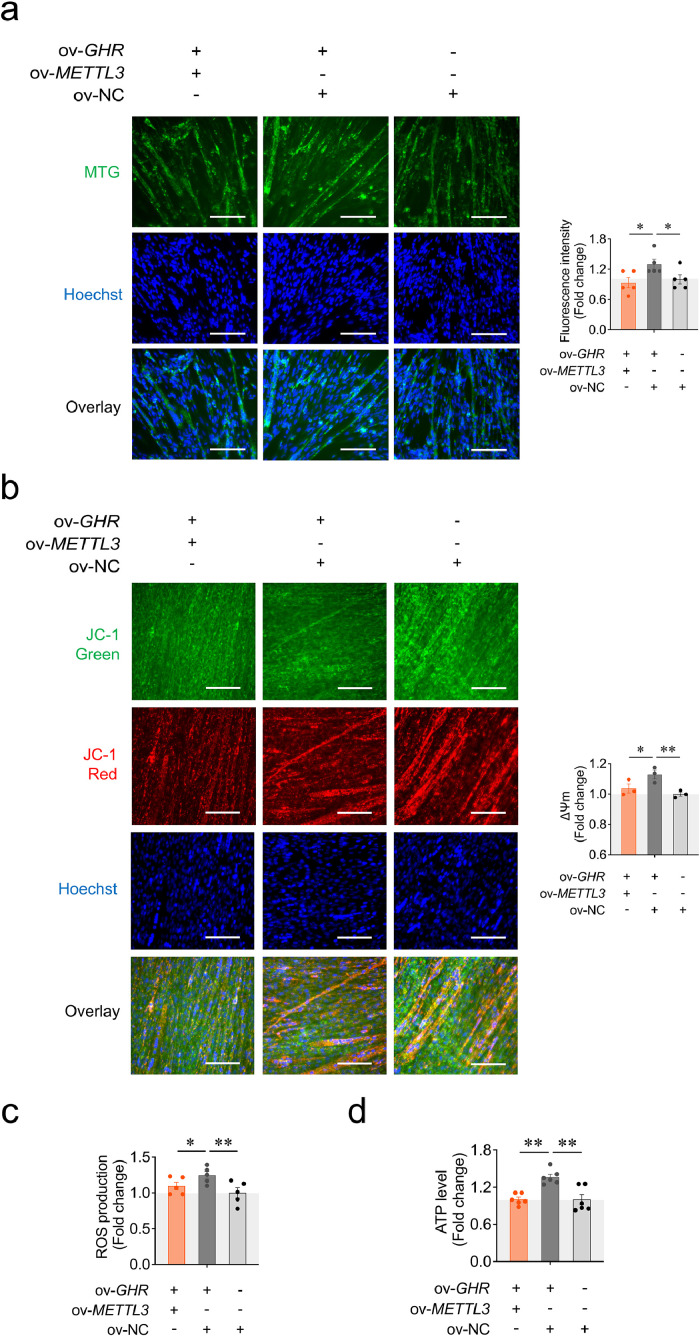
METTL3-dependent m^6^A modification of *GHR* mRNA regulates mitochondrial function during CPM differentiation. (a) Mitochondrial mass was measured by the fluorescence of MitoTracker-Green (MTG) at 48 h after co-transfection with pcDNA3.1-*METTL3*, pcDNA3.1-*GHR* and pcDNA3.1 (*n* = 5), bar 100 μm. (b) Mitochondrial membrane potential (ΔΨm) was assessed by JC-1 fluorescence 48 h after co-transfection with pcDNA3.1-*METTL3*, pcDNA3.1-*GHR*, and pcDNA3.1 (*n* = 3). Scale bar: 100 μm. (c) Reactive oxygen species (ROS) production was measured by DCF fluorescence 48 h after co-transfection with pcDNA3.1-*METTL3*, pcDNA3.1-*GHR*, and pcDNA3.1 (*n* = 5). (d) ATP levels were measured 48 h after co-transfection with pcDNA3.1-*METTL3*, pcDNA3.1-*GHR*, and pcDNA3.1 (*n* = 6). Data are shown as mean ± SEM, *p < 0.05, **p < 0.01.

Mitochondrial biogenesis is essential for maintaining normal mitochondrial oxidative phosphorylation (OXPHOS), raising the question of whether METTL3-mediated m⁶A modification of *GHR* mRNA affects mitochondrial function during CPM differentiation. To address this, we first assessed mitochondrial mass using MTG staining. A reduction in mitochondrial mass was observed in the co-transfection group compared to the *GHR* overexpression group (Fig. 4**a**). We then evaluated mitochondrial function by measuring mitochondrial membrane potential (ΔΨm), intracellular ATP levels (via a luminescence-based assay), and ROS production. The ΔΨm and ATP level as well as ROS production were all significantly decreased in the co-transfection group compared with the *GHR* overexpression group (Fig. 4**b-d**). Similarly, overexpression of *METTL3* alone reduced the mitochondrial mass of CPM, and decreased the ΔΨm and ATP level, but had no effect on ROS production (Fig. S3a-d). These results suggest that METTL3-dependent m^6^A modification of *GHR* mRNA impairs mitochondrial function during myoblast differentiation.

### Exploration of potential m^6^A reader protein related to METTL3-dependent m^6^A modification of GHR mRNA during CPM differentiation

**Fig. 5:**
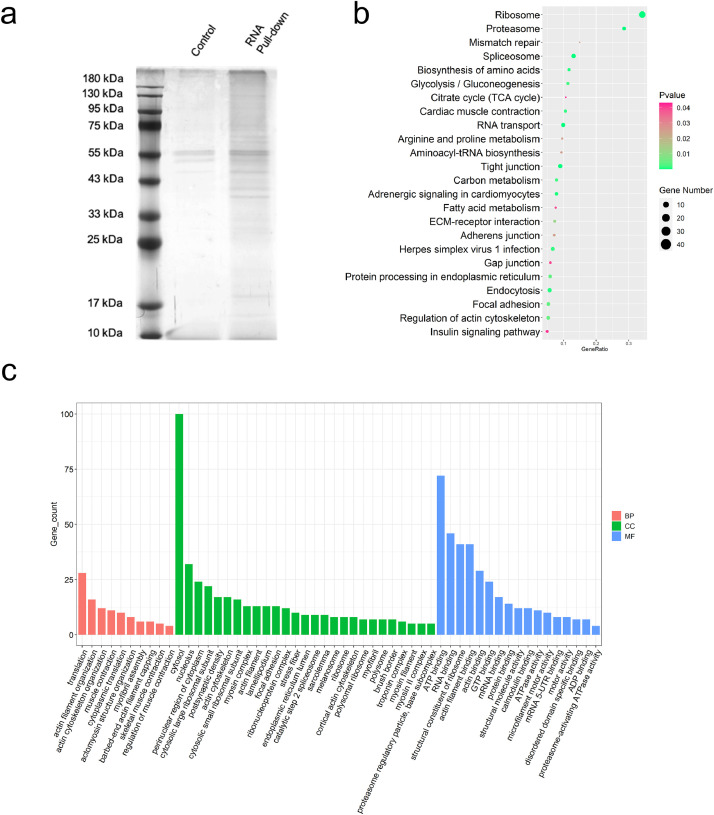
Exploration of potential reader protein involved in METTL3-dependent m^6^A modification of *GHR* mRNA during CPM differentiation. (a) Silver staining revealed the total protein precipitated after *GHR* mRNA pull-down. (b) KEGG pathways analysis of differentially expressed protein between *GHR* mRNA pull-down group and control group. (c) GO functions analysis of differentially expressed protein between *GHR* mRNA pull-down group and control group. BP: Biological Process, CC: Cellular Component, MF: Molecular Function.

We next attempted to uncover the potential m^6^A reader proteins that bind to *GHR* mRNA by RNA pull-down coupled with mass spectrometry experiments (Fig. 5**a**). A total of 435 proteins were found to specifically bind to *GHR* mRNA (Table S3). The Kyoto Encyclopedia of Genes and Genomes (KEGG) pathway analysis revealed that these specific proteins were mainly enriched in the ribosome, spliceosome, tight junction, RNA transport and endocytosis (Fig. 5**b**). Meanwhile, the gene ontology (GO) functional analysis revealed that these specific proteins enriched biological processes mainly in translation and muscle contraction; molecular functions were mainly in ATP binding and RNA binding; cell components were mainly in cytosol and nucleolus (Fig. 5**c**). Among these, *METTL3* along with three methylated reader proteins hnRNPR, hnRNPA3 and hnRNPM were found to be precipitated (Fig. S4a-d). Taken together, these results suggest that hnRNPR, hnRNPA3 and hnRNPM might be potential m^6^A reader proteins involved in METTL3-dependent m^6^A modification of *GHR* mRNA during myoblast differentiation.

## Discussion

The GH-GHR-IGF1 axis has been studied for over a century, with research primarily focused on the treatment of growth-related disorders (Ranke and Wit, 2018). In our previous research, we demonstrated that GHR-IGF1 regulates mitochondrial function and biogenesis via the PI3K/AKT/CREB pathway, thereby promoting myogenic differentiation (Hu et al., 2023). As the central hub of cellular energy metabolism, mitochondria play a vital role in myoblast differentiation and muscle development. In the early stages of myogenic differentiation, cells first remove dysfunctional mitochondria through mitophagy, followed by the induction of mitochondrial biogenesis to produce a large population of new mitochondria that meet the heightened energy demands (Sin et al., 2016). PGC1α, a key regulator of mitochondrial biogenesis, exhibits a pronounced role in promoting myogenic differentiation in skeletal muscle (Beltrà et al., 2022). Furthermore, mitochondrial dysfunction is closely associated with muscle aging and the progression of Duchenne muscular dystrophy (Pääsuke et al., 2016; Vila et al., 2017). We revealed that METTL3-dependent m^6^A modification down-regulates *GHR* mRNA expression to impair mitochondrial function by inhibiting mitochondrial biogenesis during myoblast differentiation. This disruption may ultimately hinder the differentiation process.

RNA post-transcriptional modifications of RNA are widely present in eukaryotic genomes and play an important role in many biological processes such as development, reproduction and disease (Wu et al., 2018; Tong et al., 2018; Cheng et al., 2021). To date, more than 100 chemical modifications have been identified in the cellular RNA of eukaryotic genomes, including N6-methyladenosine (m^6^A), N7-methylguanine (m^7^G) and 5-methylcytosine (m^5^C). While m^6^A modification is the most common RNA modification in eukaryotes which has been reported to play a pivotal role in animal muscle development (Li et al., 2021). In poultry, cycloleucine consistently reduces the m^6^A modification levels in myoblast cells, thereby inhibiting myoblast proliferation and suppressing myotube formation (Wang et al., 2022b). Conversely, low betaine concentrations increase the level of m^6^A modification, promoting myotube formation but inhibiting cell proliferation in vitro (Wang et al., 2022a). These results indicate that m^6^A modification is associated with chicken muscle development. However, the relationship between m⁶A modification and muscle development is complex. The m^6^A modification is modified by the m^6^A methyltransferases, such as METTL3, METTL14, and METTL16 (Jiang et al., 2021). Studies have shown that METTL3 promotes the myogenic differentiation of goat skeletal muscle satellite cells by regulating the expression of *MEF2C* (Zhao et al., 2023). In avian species, METTL14 facilitates the differentiation of duck embryonic muscle cells by modulating the expression of miR-133b (Jiang et al., 2025). In contrast, METTL16 promotes the proliferation of chicken myoblasts but inhibits their differentiation (Liu et al., 2024a), which may be due to differences in the target genes modified by distinct methyltransferases. On the other hand, the GH-GHR-IGF1 axis in the liver has been demonstrated to exhibit great complexity in response to muscle development. There is evidence that IGFBP2 and METTL3 were identified as key factors regulating m^6^A of *NPR3* and *GHR* predicted by machine learning in synovial fibroblasts (Xiao et al., 2022). *GHR* has direct interaction with the m^6^A protein demonstrated by the m^6^A RIP-qRT-PCR experiment in gastric cancer cell lines (Jiang et al., 2022). In this study, we identified that the m⁶A modification site *GHR*-139 on *GHR* mRNA is regulated by METTL3, consistent with findings from previous research. Specifically, METTL3-mediated m⁶A modification influences mitochondrial function by modulating *GHR* expression in myoblasts. Previous studies have found that METTL3 and YTHDF2 can synergistically modify *PGC-1α* mRNA, mediating its degradation, thereby impairing mitochondrial function and enhancing inflammatory responses (Zhang et al., 2021). Most existing research on m⁶A modification and muscle development focuses on direct gene regulatory mechanisms, with limited attention to mitochondrial function (Yu et al., 2022). Our findings provide a new research avenue for elucidating the role of METTL3-mediated m^6^A modification in muscle development. This insight provides a theoretical basis for developing RNA m⁶A-targeted strategies to restore mitochondrial function and promote muscle growth. However, it is important to note that this study focuses exclusively on METTL3-mediated m⁶A modification of *GHR*. Further investigation is required to determine whether METTL3 also affects mitochondrial function through additional pathways during myogenic differentiation.

The results of m^6^A modification are intricate and variable which are determined by the dynamic balance of m^6^A-specific reader proteins and m^6^A modified mRNAs (Wang et al., 2014; Zaccara et al., 2019). At present, numerous m^6^A-specific reading proteins have been revealed to regulate m^6^A modification in different ways, including YTHDF1/2/3 (Ye et al., 2020), YTHDC1/2 (Xiao et al., 2016; Shi et al., 2017; Kretschmer et al., 2018; Fei et al., 2020), hnRNPA2B1 and hnRNPC (Yue et al., 2015; Wang et al., 2015), IGF2BP1/2/3 (Huang et al., 2018) as well as eIF3 (Zaccara et al., 2019). Generally, YTHDF1/2/3, IGF2BP1/2/3 and eIF3 regulate mRNA stability and translation, YTHDC1/2 regulates mRNA export, as well as hnRNPA2B1 and hnRNPC regulate primary microRNA processing and mRNA splicing. In this study, we revealed that METTL3-dependent m^6^A modification down-regulated *GHR* mRNA and protein expression without impinging on its stability by RT-qPCR, Western blots and ACTD experiments. According to the above results, we then uncovered three m^6^A reader proteins hnRNPR, hnRNPA3 and hnRNPM precipitated by RNA pull-down experiments.

HnRNPs mainly play their roles in transcriptional and post-transcriptional regulation of gene expression, including RNA splicing, polyadenylation, capping and translation (Keene, 2007; Glisovic et al., 2008). Each hnRNP family member contains at least one RNA binding domain (RBD), such as an RNA recognition motif (RRM), a K-homology (KH) domain or an arginine/glycine-rich domain (He and Smith, 2009). hnRNPA2B1 has been reported to recognize m^6^A modification sites of mRNAs to progress post-transcriptional modification resemble alternative splicing, or m^6^A modified miRNA sequence to influence primary miRNA processing (Alarcón et al., 2015). While m^6^A modification also influences the RNA secondary structure, hnRNPC has been demonstrated to recognize m^6^A modification sites to regulate mRNA abundance and splicing, which is called "the m^6^A-switch" (Liu et al., 2015). Studies have shown that hnRNPR and hnRNPA2B1 bind to and stabilize *ASCL1* mRNA in a m⁶A-dependent manner, thereby promoting the progression of neuroblastoma (Hu et al., 2024). In addition, hnRNPM appears to play a critical role in the regulation of m⁶A-mediated alternative splicing of *HO-1* mRNA (Su et al., 2024). Similarly, hnRNPA3 may function as a m⁶A reader protein that recognizes and modulates the alternative splicing of the oncogenic fusion gene *AML1/ETO* pre-mRNA, thus contributing to the progression of acute myeloid leukemia (Liu et al., 2024b). The roles of these three m⁶A reader proteins in muscle development have not been reported in previous studies. Based on our findings, we hypothesize that m⁶A modification may influence *GHR* mRNA expression by regulating its splicing via hnRNP proteins. However, the specific molecular mechanisms underlying this regulation require further validation.

## Conclusion

**Fig. 6:**
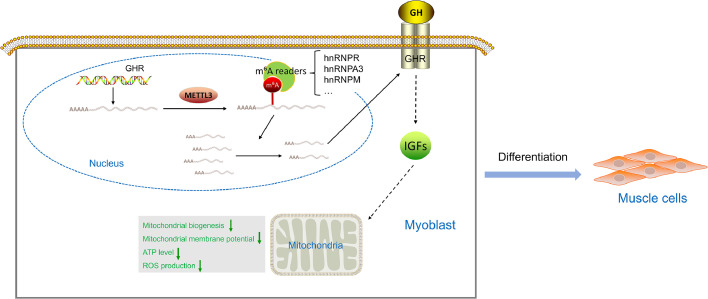
The schematic diagram illustrates the mechanisms of METTL3-dependent m^6^A modification in *GHR* mRNA regulation during myoblast differentiation in chickens.

In conclusion, our data corroborate that METTL3-dependent m^6^A modification down-regulated *GHR* mRNA expression to impair mitochondrial function by inhibiting mitochondrial biogenesis during myoblast differentiation (Fig. 6). We conducted an in-depth investigation into the relationship between m^6^A-modified *GHR* mRNA and mitochondrial function during myogenic differentiation, offering new insights into m^6^A modification and muscle development. This study also identifies potential molecular targets for treating muscular diseases associated with mitochondrial dysfunction. In the field of poultry, this discovery lays a theoretical foundation and supports the selection and breeding of desirable meat traits in chickens.

## Declaration of competing interest

The authors declare no conflicts of interest.

## Appendix Supplementary materials

Fig. S1 The peaks of m^6^A modification in *GHR* mRNA and the verification of its methyltransferase activity. (a) The m^6^A modification peaks via meRIP-Seq on *GHR* mRNA as visualized by IGV. (b) The *METTL3* mRNA expression was measured by RT-qPCR after transfection with pcDNA3.1-*METTL3* and pcDNA3.1 (*n* = 4). (c) Relative luciferase activities of CPM co-transfected with plasmids containing wild-type or mutant *GHR* mRNA and *METTL14* cDNA. Firefly luciferase activities were measured and normalized to renilla luciferase activity (*n* = 6). (d) Relative luciferase activities of CPM co-transfected with plasmids containing wild-type or mutant *GHR* mRNA and *METTL16* cDNA. Firefly luciferase activities were measured and normalized to renilla luciferase activity (*n* = 6). Data are shown as mean ± SEM, ***p* < 0.01, ns means no significant. Image, image 1

Fig. S2 *METTL3* overexpression suppressed the expression of mitochondrial genes and genes related to mitochondrial biogenesis during CPM differentiation. (a) The *METTL3* mRNA expression was measured by RT-qPCR at 48 h after transfection with pcDNA3.1-*METTL3* and pcDNA3.1 (*n* = 3). (b) The *GHR* mRNA expression was measured by RT-qPCR at 48 h after transfection with pcDNA3.1-*METTL3* and pcDNA3.1 (*n* = 3). (c) The mtDNA copy number was measured by qPCR at 48 h after transfection with pcDNA3.1-*METTL3* and pcDNA3.1 (*n* = 3). (d) The mtDNA transcription was measured by RT-qPCR at 48 h after transfection with pcDNA3.1-*METTL3* and pcDNA3.1 (*n* = 3). (e) The *PGC1α, NRF1, TFAM* mRNA expression was measured by RT-qPCR at 48 h after transfection with pcDNA3.1-*METTL3* and pcDNA3.1 (*n* = 3). (f, g) The PGC1α, NRF1, and TOMM20 protein expression was measured by western blots at 48 h after transfection with pcDNA3.1-*METTL3* and pcDNA3.1 (*n* = 2). Data are shown as mean ± SEM, **p* < 0.05, ***p* < 0.01. Image, image 2

Fig. S3 *METTL3* overexpression regulates mitochondrial function during CPM differentiation. (a) Mitochondrial mass was measured by the fluorescence of MitoTracker-Green (MTG) at 48 h after transfection with pcDNA3.1-*METTL3* and pcDNA3.1 (*n* = 4), bar 100 μm. (b) ΔΨm was measured by the fluorescence of JC-1 at 48 h after transfection with pcDNA3.1-*METTL3* and pcDNA3.1 (*n* = 4), bar 100 μm. (c) Reactive oxygen species production was measured by the fluorescence of DCF at 48 h after transfection with pcDNA3.1-*METTL3* and pcDNA3.1 (*n* = 4). (d) ATP level was measured at 48 h after transfection with pcDNA3.1-*METTL3* and pcDNA3.1 (*n* = 4). Data are shown as mean ± SEM, **p* < 0.05, ***p* < 0.01, ns means no significant. Image, image 3

Fig. S4 The mass spectrometry sequence diagram of potential reader protein involved in METTL3-dependent m^6^A modification of *GHR* mRNA. (a) METTL3 mass spectrum sequence diagram. (b) hnRNPR mass spectrum sequence diagram. (c) hnRNPA3 mass spectrum sequence diagram. (d) hnRNPM mass spectrum sequence diagram. Image, image 4

Image, application 5
