# Supplementary figures and images for: METTL3-dependent m6A modification of GHR mRNA regulates mitochondrial function through mitochondrial biogenesis during myoblast differentiation
Source: Poult Sci. 2025 Apr 25;104(7):105216. doi: 10.1016/j.psj.2025.105216 (PMC12138421; doi:10.1016/j.psj.2025.105216)

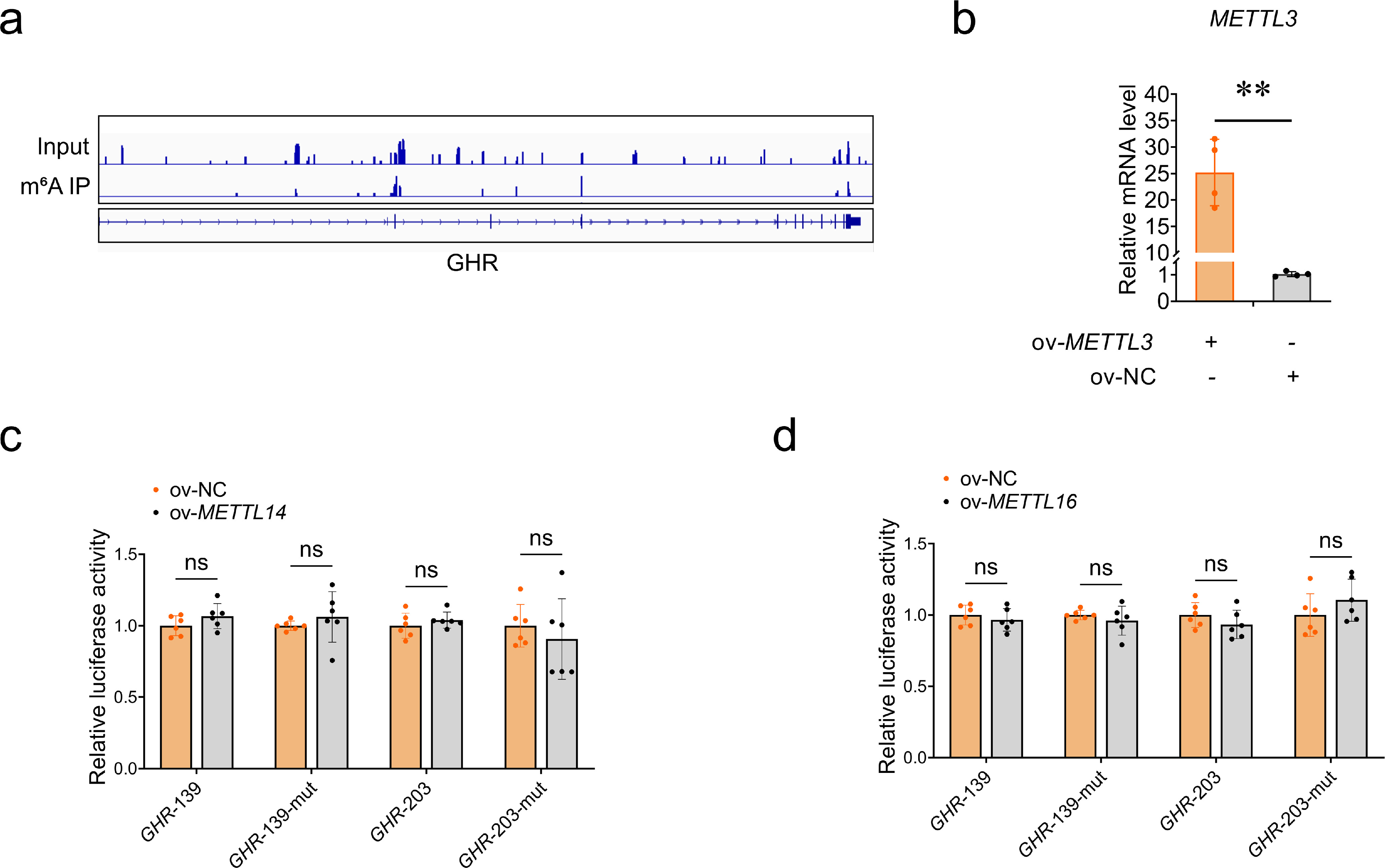

Supplement: Fig. S1 — The peaks of m6A modification in GHR mRNA and the verification of its methyltransferase activity. (a) The m6A modification peaks via meRIP-Seq on GHR mRNA as visualized by IGV. (b) The METTL3 mRNA expression was measured by RT-qPCR after transfection with pcDNA3.1-METTL3 and pcDNA3.1 (n = 4). (c) Relative luciferase activities of CPM co-transfected with plasmids containing wild-type or mutant GHR mRNA and METTL14 cDNA. Firefly luciferase activities were measured and normalized to renilla luciferase activity (n = 6). (d) Relative luciferase activities of CPM co-transfected with plasmids containing wild-type or mutant GHR mRNA and METTL16 cDNA. Firefly luciferase activities were measured and normalized to renilla luciferase activity (n = 6). Data are shown as mean ± SEM, **p < 0.01, ns means no significant. [file mmc1.jpg]

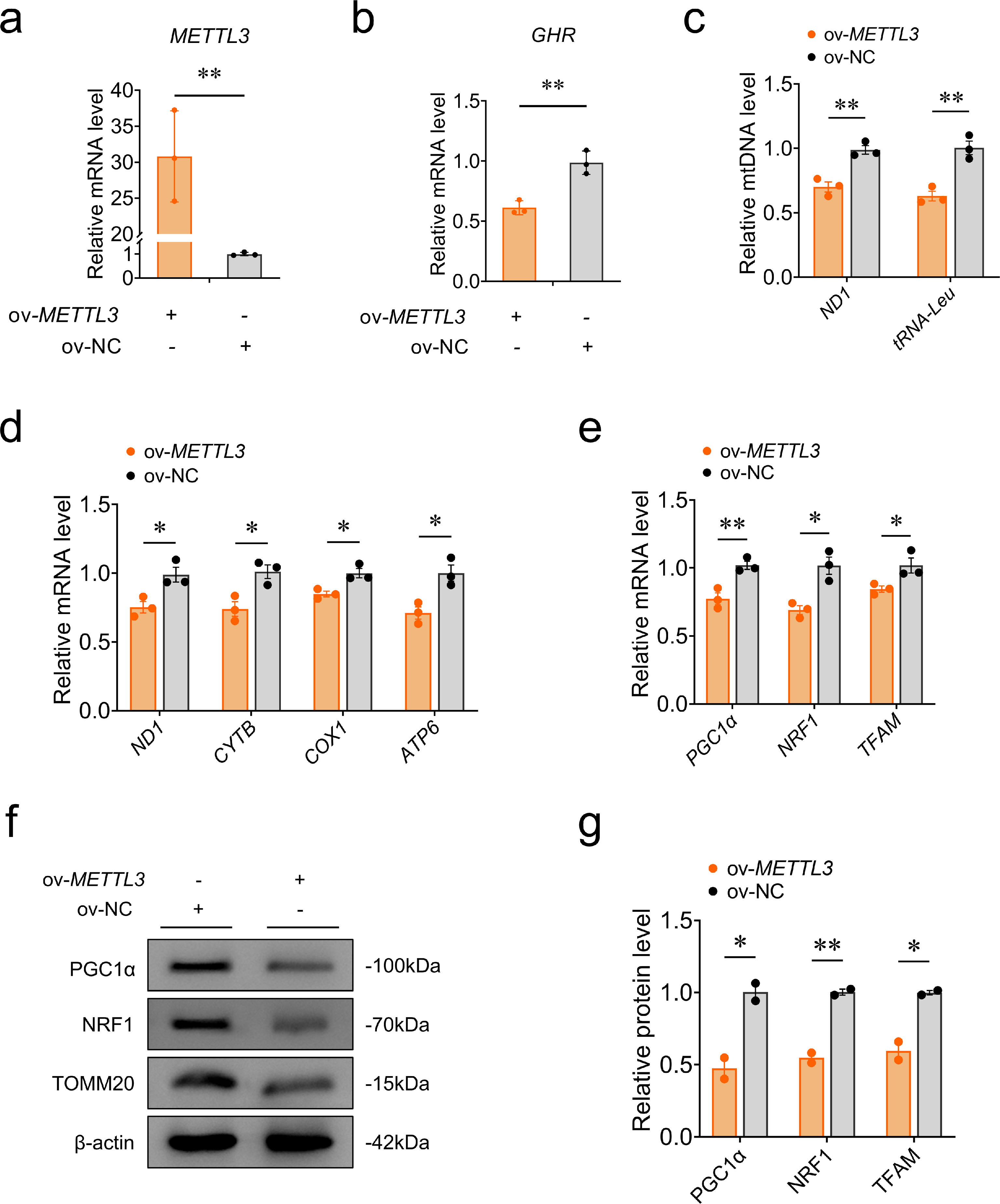

Supplement: Fig. S2 — METTL3 overexpression suppressed the expression of mitochondrial genes and genes related to mitochondrial biogenesis during CPM differentiation. (a) The METTL3 mRNA expression was measured by RT-qPCR at 48 h after transfection with pcDNA3.1-METTL3 and pcDNA3.1 (n = 3). (b) The GHR mRNA expression was measured by RT-qPCR at 48 h after transfection with pcDNA3.1-METTL3 and pcDNA3.1 (n = 3). (c) The mtDNA copy number was measured by qPCR at 48 h after transfection with pcDNA3.1-METTL3 and pcDNA3.1 (n = 3). (d) The mtDNA transcription was measured by RT-qPCR at 48 h after transfection with pcDNA3.1-METTL3 and pcDNA3.1 (n = 3). (e) The PGC1α, NRF1, TFAM mRNA expression was measured by RT-qPCR at 48 h after transfection with pcDNA3.1-METTL3 and pcDNA3.1 (n = 3). (f, g) The PGC1α, NRF1, and TOMM20 protein expression was measured by western blots at 48 h after transfection with pcDNA3.1-METTL3 and pcDNA3.1 (n = 2). Data are shown as mean ± SEM, *p < 0.05, **p < 0.01. [file mmc2.jpg]

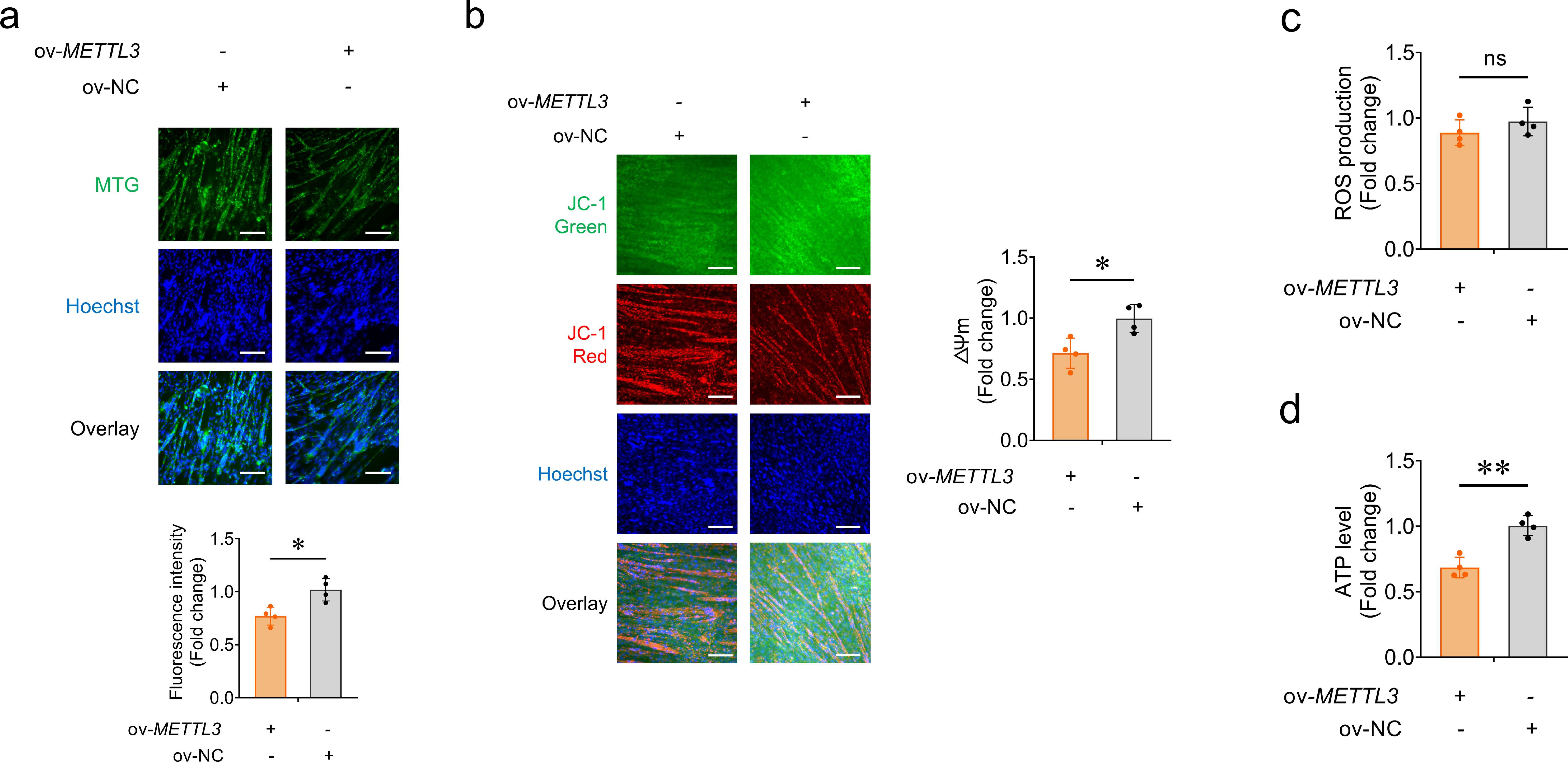

Supplement: Fig. S3 — METTL3 overexpression regulates mitochondrial function during CPM differentiation. (a) Mitochondrial mass was measured by the fluorescence of MitoTracker-Green (MTG) at 48 h after transfection with pcDNA3.1-METTL3 and pcDNA3.1 (n = 4), bar 100 μm. (b) ΔΨm was measured by the fluorescence of JC-1 at 48 h after transfection with pcDNA3.1-METTL3 and pcDNA3.1 (n = 4), bar 100 μm. (c) Reactive oxygen species production was measured by the fluorescence of DCF at 48 h after transfection with pcDNA3.1-METTL3 and pcDNA3.1 (n = 4). (d) ATP level was measured at 48 h after transfection with pcDNA3.1-METTL3 and pcDNA3.1 (n = 4). Data are shown as mean ± SEM, *p < 0.05, **p < 0.01, ns means no significant. [file mmc3.jpg]

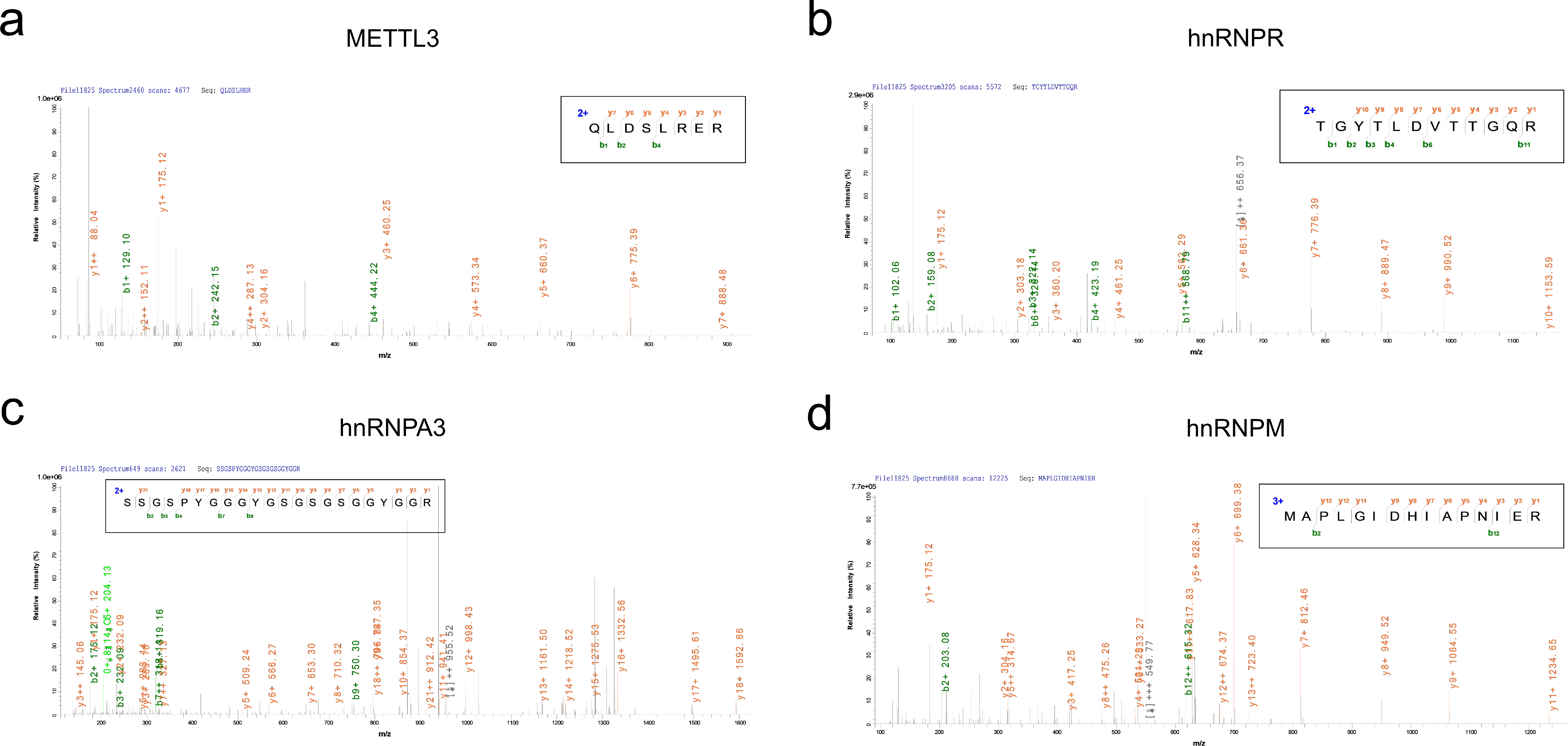

Supplement: Fig. S4 — The mass spectrometry sequence diagram of potential reader protein involved in METTL3-dependent m6A modification of GHR mRNA. (a) METTL3 mass spectrum sequence diagram. (b) hnRNPR mass spectrum sequence diagram. (c) hnRNPA3 mass spectrum sequence diagram. (d) hnRNPM mass spectrum sequence diagram. [file mmc4.jpg]
