# Supplementary material for: METTL3-dependent m6A modification of GHR mRNA regulates mitochondrial function through mitochondrial biogenesis during myoblast differentiation
Source: Poult Sci. 2025 Apr 25;104(7):105216. doi: 10.1016/j.psj.2025.105216 (PMC12138421; doi:10.1016/j.psj.2025.105216)
Supplement: Supplementary file 5 [file mmc5.docx]

Supplementary materials

**Table S1.** The sequences of primers for qRT-PCR

| Gene | Primer sequence, 5’ to 3’ | Size, bp | Notes |
| --- | --- | --- | --- |
| *GH* | F-TGCAATACCTAAGCAAGGTGTTCA  R-CAGGGCTTGGATCCCTTCTT | 101 | qRT-PCR |
| *GHR*  *METTL3* | F-GCGTGTTCAGGAGCAAAGCT  R-TGGGACAGGCATTTCCATACTT  F- GGTCCCACCATCGTCACCTA  R- CGCCTGCTTACGGGATTTCT | 121  189 | qRT-PCR  qRT-PCR |
| *IGF1* | F-AGTTCGTATGTGGAGACAGAGGC  R-CCAGCCTCCTCAGGTCACAAC | 129 | qRT-PCR |
| *IGF2* | F-AGACCAGTGGGACGAAATAACA  R-CACGCTCTGACTTGACGGAC | 121 | qRT-PCR |
| *PGC1α* | F-ACTACGAACGATGACCCTCC  R-CCCTTGGGATCATTTGGAGACT | 173 | qRT-PCR |
| *NRF1* | F-ACGAGGACTCACCTTCCTCA  R-TGTGGTCGCTTCCGTTTCTT | 163 | qRT-PCR |
| *TFAM* | F-GACCTCGAAGTGGCTTCAAC  R-GAGCAAGCTGAAGGTATGGCT | 144 | qRT-PCR |
| *ND1* | F-ACCCAAGAGCCCATCTACCT  R-GTCCGGCGGCATATTCTACA | 154 | qPCR, qRT-PCR |
| *CYTB* | F-CAGCAGACACATCCCTAGCC  R-GAAGAATGAGGCGCCGTTTG | 104 | qRT-PCR |
| *COX1* | F-ACTACTTACCGACCGCAACC  R-CCGAAACCTGGGAGGATGAG | 132 | qRT-PCR |
| *ATP6* | F-TACAGCCACAATCGCCCTAC  R-AGGACGAAGACGTAGGCTTG | 123 | qRT-PCR |
| *tRNA-Leu* | F-GCTCGGCAAATGCAAAAGG  R-AGGATTTGAACCTCTGGATAAAGGG | 50 | qPCR |
| *β-actin* | F-GATATTGCTGCGCTCGTTG  R-TTCAGGGTCAGGATACCTCTTT | 178 | qRT-PCR |

**Table S2.** Primers for PCR

| Gene | Primer sequence, 5’ to 3’ | Size, bp |
| --- | --- | --- |
| *GHR-*site139-203 | F- GCCCCTGCTGACATTTGAGAAT  R- ATCGAGGTGTAGGATGTGTTGA | 304 |
| *GHR-*site 385 | F- AAGTATTTGACGAAAAGTGTTTCAG  R- CCTGGGTTCTAACTCCTTCCATT | 203 |

**Table S3.** Specific protein summary of GHR pull down

| \| Number \| Protein access \| Protein description \| \| --- \| --- \| --- \| \| 1 \| tr\|A0A1D5PM19\|A0A1D5PM19_CHICK \| Myosin-9 OS=Gallus gallus OX=9031 GN=MYH9 PE=3 SV=2 \| \| 2 \| tr\|Q789A6\|Q789A6_CHICK \| Nonmuscle myosin heavy chain OS=Gallus gallus OX=9031 GN=MYH10 PE=2 SV=1 \| \| 4 \| sp\|P68034\|ACTC_CHICK \| Actin, alpha cardiac muscle 1 OS=Gallus gallus OX=9031 GN=ACTC1 PE=3 SV=1 \| \| 5 \| tr\|F1P476\|F1P476_CHICK \| Actin, aortic smooth muscle OS=Gallus gallus OX=9031 GN=ACTA2 PE=3 SV=2 \| \| 6 \| sp\|P68139\|ACTS_CHICK \| Actin, alpha skeletal muscle OS=Gallus gallus OX=9031 GN=ACTA1 PE=1 SV=1 \| \| 9 \| tr\|A0A1D5PGL4\|A0A1D5PGL4_CHICK \| Uncharacterized protein OS=Gallus gallus OX=9031 GN=MYO18A PE=3 SV=2 \| \| 10 \| tr\|F1NJ08\|F1NJ08_CHICK \| Vimentin OS=Gallus gallus OX=9031 GN=VIM PE=3 SV=1 \| \| 12 \| tr\|A0A1D5PGD5\|A0A1D5PGD5_CHICK \| Collagen alpha-3(VI) chain OS=Gallus gallus OX=9031 GN=COL6A3 PE=4 SV=2 \| \| 13 \| tr\|A0A1D5P287\|A0A1D5P287_CHICK \| Myosin-11 OS=Gallus gallus OX=9031 GN=MYH11 PE=3 SV=2 \| \| 14 \| tr\|Q05705\|Q05705_CHICK \| Beta-tropomyosin OS=Gallus gallus OX=9031 GN=BRT-2 PE=2 SV=1 \| \| 16 \| tr\|A0A1L1RM08\|A0A1L1RM08_CHICK \| Uncharacterized protein OS=Gallus gallus OX=9031 GN=MYL12A PE=4 SV=1 \| \| 19 \| tr\|A0A3Q3ACB9\|A0A3Q3ACB9_CHICK \| Tropomyosin beta chain OS=Gallus gallus OX=9031 GN=TPM2 PE=3 SV=1 \| \| 21 \| tr\|A0A1D5NWY3\|A0A1D5NWY3_CHICK \| Unconventional myosin-Ic OS=Gallus gallus OX=9031 GN=MYO1C PE=3 SV=1 \| \| 23 \| tr\|A0A1D5NXV1\|A0A1D5NXV1_CHICK \| Tubulin alpha chain OS=Gallus gallus OX=9031 GN=LOC100857858 PE=3 SV=2 \| \| 24 \| sp\|O93510\|GELS_CHICK \| Gelsolin OS=Gallus gallus OX=9031 GN=GSN PE=2 SV=1 \| \| 28 \| tr\|A0A1D5NT47\|A0A1D5NT47_CHICK \| Uncharacterized protein OS=Gallus gallus OX=9031 GN=TPM4 PE=3 SV=2 \| \| 32 \| tr\|A0A3Q3AR57\|A0A3Q3AR57_CHICK \| C3H1-type domain-containing protein OS=Gallus gallus OX=9031 GN=HELZ2 PE=4 SV=1 \| \| 34 \| tr\|A0A3Q2TW07\|A0A3Q2TW07_CHICK \| Fibronectin OS=Gallus gallus OX=9031 GN=FN1 PE=4 SV=1 \| \| 35 \| tr\|A0A1D5NYM1\|A0A1D5NYM1_CHICK \| ATP-dependent 6-phosphofructokinase OS=Gallus gallus OX=9031 GN=PFKP PE=3 SV=1 \| \| 37 \| tr\|F1NTJ5\|F1NTJ5_CHICK \| Uncharacterized protein OS=Gallus gallus OX=9031 GN=MYO1B PE=3 SV=4 \| \| 38 \| tr\|F1NW97\|F1NW97_CHICK \| Tubulin alpha chain OS=Gallus gallus OX=9031 GN=TUBA4B PE=3 SV=4 \| \| 39 \| sp\|P21868\|CSK21_CHICK \| Casein kinase II subunit alpha OS=Gallus gallus OX=9031 GN=CSNK2A1 PE=2 SV=1 \| \| 40 \| tr\|F1NWX0\|F1NWX0_CHICK \| Tubulin alpha chain OS=Gallus gallus OX=9031 GN=LOC425049 PE=3 SV=3 \| \| 42 \| tr\|A0A1D5PAB0\|A0A1D5PAB0_CHICK \| Unconventional myosin-Va OS=Gallus gallus OX=9031 GN=MYO5A PE=3 SV=2 \| \| 43 \| tr\|A0A1D5PTF4\|A0A1D5PTF4_CHICK \| LIM zinc-binding domain-containing protein OS=Gallus gallus OX=9031 GN=LIMA1 PE=4 SV=2 \| \| 44 \| tr\|Q5ZLY3\|Q5ZLY3_CHICK \| Uncharacterized protein OS=Gallus gallus OX=9031 GN=TMOD3 PE=2 SV=1 \| \| 45 \| tr\|A0A1D5PA34\|A0A1D5PA34_CHICK \| Tubulin alpha chain OS=Gallus gallus OX=9031 GN=TUBA3E PE=3 SV=1 \| \| 47 \| tr\|H9L0X6\|H9L0X6_CHICK \| 2~-5~ oligoadenylate synthase OS=Gallus gallus OX=9031 GN=OASL PE=3 SV=3 \| \| 48 \| tr\|A0A140KDZ9\|A0A140KDZ9_CHICK \| Myxocirus-resistance protein (Fragment) OS=Gallus gallus OX=9031 GN=Mx1 PE=2 SV=1 \| \| 49 \| tr\|A0A1D5NZ71\|A0A1D5NZ71_CHICK \| J domain-containing protein OS=Gallus gallus OX=9031 GN=DNAJC13 PE=4 SV=2 \| \| 50 \| tr\|A0A1D5PEM4\|A0A1D5PEM4_CHICK \| Myosin light polypeptide 6 OS=Gallus gallus OX=9031 GN=MYL6 PE=4 SV=3 \| \| 51 \| tr\|A0A1D5P8I8\|A0A1D5P8I8_CHICK \| Collagen alpha-1(XII) chain OS=Gallus gallus OX=9031 GN=COL12A1 PE=4 SV=2 \| \| 53 \| sp\|P00356\|G3P_CHICK \| Glyceraldehyde-3-phosphate dehydrogenase OS=Gallus gallus OX=9031 GN=GAPDH PE=2 SV=3 \| \| 55 \| tr\|A0A1X9WEL5\|A0A1X9WEL5_CHICK \| Elongation factor Tu OS=Gallus gallus OX=9031 GN=TUFM PE=2 SV=1 \| \| 56 \| tr\|A0A1D5P2H5\|A0A1D5P2H5_CHICK \| Uncharacterized protein OS=Gallus gallus OX=9031 GN=CALM2 PE=4 SV=2 \| \| 57 \| tr\|R4GGF3\|R4GGF3_CHICK \| SCP domain-containing protein OS=Gallus gallus OX=9031 GN=GLIPR2 PE=4 SV=1 \| \| 58 \| tr\|A0A3Q3A7P6\|A0A3Q3A7P6_CHICK \| 40S ribosomal protein S15a OS=Gallus gallus OX=9031 GN=RPS15A PE=3 SV=1 \| \| 59 \| tr\|Q5ZII1\|Q5ZII1_CHICK \| Ribos_L4_asso_C domain-containing protein OS=Gallus gallus OX=9031 GN=RCJMB04_25p23 PE=2 SV=1 \| \| 61 \| tr\|A0A1D5PGL5\|A0A1D5PGL5_CHICK \| Uncharacterized protein OS=Gallus gallus OX=9031 GN=DOCK7 PE=3 SV=2 \| \| 62 \| tr\|F1NCG5\|F1NCG5_CHICK \| Uncharacterized protein OS=Gallus gallus OX=9031 GN=LRRFIP1 PE=3 SV=4 \| \| 63 \| tr\|A0A1D5P5X9\|A0A1D5P5X9_CHICK \| Elongation factor 1-alpha OS=Gallus gallus OX=9031 GN=EEF1A1 PE=3 SV=1 \| \| 64 \| tr\|A0A1D5NTV3\|A0A1D5NTV3_CHICK \| Acetyl-CoA carboxylase OS=Gallus gallus OX=9031 GN=ACACA PE=4 SV=1 \| \| 65 \| tr\|A0A1D5PH79\|A0A1D5PH79_CHICK \| Uncharacterized protein OS=Gallus gallus OX=9031 GN=LOC107049991 PE=4 SV=2 \| \| 66 \| tr\|R4GH27\|R4GH27_CHICK \| WH2 domain-containing protein OS=Gallus gallus OX=9031 GN=LMOD1 PE=4 SV=3 \| \| 68 \| tr\|A0A3S5ZPN3\|A0A3S5ZPN3_CHICK \| Probable ATP-dependent RNA helicase DDX5 OS=Gallus gallus OX=9031 GN=DDX5 PE=3 SV=1 \| \| 69 \| tr\|A0A1D5PKK0\|A0A1D5PKK0_CHICK \| Pyrroline-5-carboxylate reductase OS=Gallus gallus OX=9031 GN=PYCR1 PE=3 SV=2 \| \| 70 \| tr\|A0A3Q2U0S7\|A0A3Q2U0S7_CHICK \| RNA helicase OS=Gallus gallus OX=9031 GN=DDX3X PE=3 SV=1 \| \| 71 \| tr\|F2Z4K7\|F2Z4K7_CHICK \| 40S ribosomal protein S3a OS=Gallus gallus OX=9031 GN=RPS3A PE=3 SV=3 \| \| 72 \| sp\|P09860\|TNNC1_CHICK \| Troponin C, slow skeletal and cardiac muscles OS=Gallus gallus OX=9031 GN=TNNC1 PE=1 SV=1 \| \| 73 \| tr\|A0A1D5PFJ6\|A0A1D5PFJ6_CHICK \| Heat shock cognate 71 kDa protein OS=Gallus gallus OX=9031 GN=HSPA8 PE=3 SV=2 \| \| 74 \| tr\|A0A1D5PWN6\|A0A1D5PWN6_CHICK \| Collagen alpha-1(VI) chain OS=Gallus gallus OX=9031 GN=COL6A1 PE=4 SV=2 \| \| 75 \| sp\|Q5ZJ56\|RL7_CHICK \| 60S ribosomal protein L7 OS=Gallus gallus OX=9031 GN=RPL7 PE=2 SV=1 \| \| 76 \| tr\|R4GIG1\|R4GIG1_CHICK \| Uncharacterized protein OS=Gallus gallus OX=9031 GN=MYH1D PE=3 SV=3 \| \| 78 \| tr\|A0A1D5PSI2\|A0A1D5PSI2_CHICK \| Inosine-5~-monophosphate dehydrogenase OS=Gallus gallus OX=9031 GN=IMPDH2 PE=3 SV=1 \| \| 79 \| tr\|A0A1D5PBF6\|A0A1D5PBF6_CHICK \| Uncharacterized protein OS=Gallus gallus OX=9031 GN=FLII PE=4 SV=2 \| \| 80 \| tr\|A0A1D5PCT4\|A0A1D5PCT4_CHICK \| Coronin OS=Gallus gallus OX=9031 GN=CORO1C PE=3 SV=1 \| \| 81 \| tr\|A0A1L1RY04\|A0A1L1RY04_CHICK \| ATP synthase subunit beta OS=Gallus gallus OX=9031 GN=ATP5B PE=3 SV=2 \| \| 82 \| sp\|P61355\|RL27_CHICK \| 60S ribosomal protein L27 OS=Gallus gallus OX=9031 GN=RPL27 PE=2 SV=2 \| \| 84 \| tr\|Q8UWG7\|Q8UWG7_CHICK \| 60S ribosomal protein L6 OS=Gallus gallus OX=9031 GN=RPL6 PE=2 SV=1 \| \| 85 \| sp\|P15988\|CO6A2_CHICK \| Collagen alpha-2(VI) chain OS=Gallus gallus OX=9031 GN=COL6A2 PE=2 SV=1 \| \| 86 \| tr\|A0A1L1RXW8\|A0A1L1RXW8_CHICK \| Troponin T, fast skeletal muscle isoforms OS=Gallus gallus OX=9031 GN=TNNT3 PE=3 SV=2 \| \| 87 \| tr\|A0A3Q2UDX1\|A0A3Q2UDX1_CHICK \| Uncharacterized protein OS=Gallus gallus OX=9031 GN=LSP1P1 PE=4 SV=1 \| \| 89 \| sp\|P41125\|RL13_CHICK \| 60S ribosomal protein L13 OS=Gallus gallus OX=9031 GN=RPL13 PE=2 SV=2 \| \| 90 \| tr\|E1BX02\|E1BX02_CHICK \| 26S proteasome non-ATPase regulatory subunit 2 OS=Gallus gallus OX=9031 GN=PSMD2 PE=3 SV=2 \| \| 91 \| tr\|F1NPA9\|F1NPA9_CHICK \| DNA-(apurinic or apyrimidinic site) lyase OS=Gallus gallus OX=9031 GN=RPS3 PE=3 SV=2 \| \| 93 \| tr\|A0A1D5PMT8\|A0A1D5PMT8_CHICK \| 60S acidic ribosomal protein P2 OS=Gallus gallus OX=9031 GN=RPLP2 PE=3 SV=1 \| \| 94 \| tr\|A0A1D5P4P9\|A0A1D5P4P9_CHICK \| HP domain-containing protein OS=Gallus gallus OX=9031 GN=SVIL PE=3 SV=1 \| \| 95 \| tr\|Q5ZHW8\|Q5ZHW8_CHICK \| Uncharacterized protein OS=Gallus gallus OX=9031 GN=RPS14 PE=2 SV=1 \| \| 96 \| tr\|E1BTG1\|E1BTG1_CHICK \| 60S ribosomal protein L12 OS=Gallus gallus OX=9031 GN=RPL12 PE=3 SV=2 \| \| 97 \| tr\|A0A1L1RWF6\|A0A1L1RWF6_CHICK \| Calponin OS=Gallus gallus OX=9031 GN=CNN3 PE=3 SV=2 \| \| 98 \| tr\|F1NW43\|F1NW43_CHICK \| Pyruvate kinase OS=Gallus gallus OX=9031 GN=PKLR PE=3 SV=2 \| \| 99 \| tr\|E1C4M0\|E1C4M0_CHICK \| 40S ribosomal protein S2 OS=Gallus gallus OX=9031 GN=RPS2 PE=3 SV=1 \| \| 101 \| tr\|A0A1D5PDA7\|A0A1D5PDA7_CHICK \| Uncharacterized protein OS=Gallus gallus OX=9031 GN=FXR1 PE=3 SV=2 \| \| 102 \| tr\|A0A1D5PBV6\|A0A1D5PBV6_CHICK \| Troponin T, cardiac muscle isoforms OS=Gallus gallus OX=9031 GN=TNNT2 PE=3 SV=2 \| \| 103 \| tr\|F1NW23\|F1NW23_CHICK \| Clathrin heavy chain OS=Gallus gallus OX=9031 GN=CLTC PE=3 SV=2 \| \| 104 \| tr\|F1NM49\|F1NM49_CHICK \| Uncharacterized protein OS=Gallus gallus OX=9031 GN=MYH15 PE=3 SV=4 \| \| 105 \| tr\|E1BY89\|E1BY89_CHICK \| 60S ribosomal protein L23 OS=Gallus gallus OX=9031 GN=RPL23 PE=3 SV=2 \| \| 106 \| sp\|P18302\|DREB_CHICK \| Drebrin OS=Gallus gallus OX=9031 GN=DBN1 PE=2 SV=4 \| \| 107 \| tr\|A0A3Q2U5W6\|A0A3Q2U5W6_CHICK \| SH3 domain-containing protein OS=Gallus gallus OX=9031 GN=LOC107050955 PE=4 SV=1 \| \| 108 \| tr\|A0A1D5PN25\|A0A1D5PN25_CHICK \| Y-box-binding protein 1 OS=Gallus gallus OX=9031 GN=YBX1 PE=4 SV=1 \| \| 109 \| tr\|A0A3Q3AUG8\|A0A3Q3AUG8_CHICK \| Uncharacterized protein OS=Gallus gallus OX=9031 GN=LOC107050717 PE=3 SV=1 \| \| 110 \| sp\|P02542\|DESM_CHICK \| Desmin OS=Gallus gallus OX=9031 GN=DES PE=1 SV=2 \| \| 112 \| tr\|F1NBA8\|F1NBA8_CHICK \| Importin N-terminal domain-containing protein OS=Gallus gallus OX=9031 GN=IPO7 PE=4 SV=3 \| \| 113 \| tr\|F1NBX4\|F1NBX4_CHICK \| 60S ribosomal protein L27a OS=Gallus gallus OX=9031 GN=RPL27A PE=3 SV=4 \| \| 114 \| tr\|A0A3Q2U5L2\|A0A3Q2U5L2_CHICK \| Protein kinase domain-containing protein OS=Gallus gallus OX=9031 GN=CSNK2A2 PE=3 SV=1 \| \| 115 \| tr\|A0A1C9KD18\|A0A1C9KD18_CHICK \| Annexin OS=Gallus gallus OX=9031 GN=ANXA2 PE=2 SV=1 \| \| 116 \| tr\|A0A1D5PD32\|A0A1D5PD32_CHICK \| RNA helicase OS=Gallus gallus OX=9031 GN=DDX17 PE=3 SV=1 \| \| 118 \| tr\|A0A1D5PIZ8\|A0A1D5PIZ8_CHICK \| Uncharacterized protein OS=Gallus gallus OX=9031 GN=IQGAP3 PE=4 SV=1 \| \| 119 \| tr\|B5BSA7\|B5BSA7_CHICK \| Antigen processing associated transporter 2 OS=Gallus gallus OX=9031 GN=TAP2 PE=2 SV=1 \| \| 120 \| tr\|A0A1L1RQ08\|A0A1L1RQ08_CHICK \| 60S ribosomal protein L7a OS=Gallus gallus OX=9031 GN=RPL7A PE=3 SV=1 \| \| 121 \| sp\|Q6ITC7\|RS13_CHICK \| 40S ribosomal protein S13 OS=Gallus gallus OX=9031 GN=RPS13 PE=2 SV=3 \| \| 123 \| tr\|E1C6J9\|E1C6J9_CHICK \| Thy-1 membrane glycoprotein OS=Gallus gallus OX=9031 GN=THY1 PE=4 SV=2 \| \| 124 \| tr\|E1BY44\|E1BY44_CHICK \| TGc domain-containing protein OS=Gallus gallus OX=9031 GN=TGM2 PE=3 SV=5 \| \| 126 \| tr\|A0A1D5NUI1\|A0A1D5NUI1_CHICK \| Uncharacterized protein OS=Gallus gallus OX=9031 GN=RPL3 PE=3 SV=2 \| \| 127 \| tr\|Q8AYP8\|Q8AYP8_CHICK \| Hexokinase OS=Gallus gallus OX=9031 GN=HK1 PE=2 SV=1 \| \| 128 \| tr\|E1C4N0\|E1C4N0_CHICK \| S10_plectin domain-containing protein OS=Gallus gallus OX=9031 GN=RPS10 PE=3 SV=2 \| \| 129 \| tr\|Q5ZLG7\|Q5ZLG7_CHICK \| ADP/ATP translocase OS=Gallus gallus OX=9031 GN=SLC25A6 PE=2 SV=1 \| \| 130 \| tr\|F1NIX0\|F1NIX0_CHICK \| 60S ribosomal protein L8 OS=Gallus gallus OX=9031 GN=RPL8 PE=3 SV=2 \| \| 131 \| tr\|A0A125RM76\|A0A125RM76_CHICK \| Interferon-induced protein with tetratricopeptide repeats 5 OS=Gallus gallus OX=9031 GN=IFIT5 PE=2 SV=1 \| \| 133 \| tr\|A0A1D5P980\|A0A1D5P980_CHICK \| Glutamine--fructose-6-phosphate transaminase (isomerizing) OS=Gallus gallus OX=9031 GN=GFPT1 PE=4 SV=2 \| \| 134 \| sp\|P47836\|RS4_CHICK \| 40S ribosomal protein S4 OS=Gallus gallus OX=9031 GN=RPS4 PE=2 SV=2 \| \| 135 \| tr\|A0A1D5NVI1\|A0A1D5NVI1_CHICK \| 60S ribosomal protein L9 OS=Gallus gallus OX=9031 GN=RPL9 PE=3 SV=1 \| \| 136 \| tr\|E1C6N0\|E1C6N0_CHICK \| MPN domain-containing protein OS=Gallus gallus OX=9031 GN=PSMD14 PE=4 SV=2 \| \| 137 \| sp\|Q5ZMD1\|1433T_CHICK \| 14-3-3 protein theta OS=Gallus gallus OX=9031 GN=YWHAQ PE=1 SV=1 \| \| 138 \| tr\|A0A1D5P1W7\|A0A1D5P1W7_CHICK \| Importin N-terminal domain-containing protein OS=Gallus gallus OX=9031 GN=KPNB1 PE=3 SV=2 \| \| 140 \| tr\|A0A1L1RL76\|A0A1L1RL76_CHICK \| 60S ribosomal protein L14 OS=Gallus gallus OX=9031 GN=RPL14 PE=3 SV=2 \| \| 141 \| tr\|F1NPM7\|F1NPM7_CHICK \| RRM domain-containing protein OS=Gallus gallus OX=9031 GN=TRA2A PE=4 SV=2 \| \| 142 \| tr\|A0A1D5PCU1\|A0A1D5PCU1_CHICK \| ADP/ATP translocase OS=Gallus gallus OX=9031 GN=SLC25A5 PE=3 SV=2 \| \| 143 \| tr\|O02869\|O02869_CHICK \| ChLAMP, g11-isoform OS=Gallus gallus OX=9031 GN=LSAMP PE=2 SV=1 \| \| 144 \| tr\|A0A1L1RZE8\|A0A1L1RZE8_CHICK \| T-complex protein 1 subunit epsilon OS=Gallus gallus OX=9031 GN=CCT5 PE=3 SV=2 \| \| 145 \| tr\|F1NHI3\|F1NHI3_CHICK \| RNA helicase OS=Gallus gallus OX=9031 GN=DHX15 PE=3 SV=3 \| \| 146 \| tr\|A0A1D5PPN9\|A0A1D5PPN9_CHICK \| Endoplasmin OS=Gallus gallus OX=9031 GN=HSP90B1 PE=3 SV=1 \| \| 147 \| tr\|A0A3Q2UN07\|A0A3Q2UN07_CHICK \| Uncharacterized protein OS=Gallus gallus OX=9031 GN=MYO10L PE=3 SV=1 \| \| 148 \| sp\|P62801\|H4_CHICK \| Histone H4 OS=Gallus gallus OX=9031 GN=H4-VII PE=1 SV=2 \| \| 149 \| tr\|A0A1D5P918\|A0A1D5P918_CHICK \| Ig-like domain-containing protein OS=Gallus gallus OX=9031 GN=NEXN PE=4 SV=2 \| \| 150 \| sp\|P0CB50\|PRDX1_CHICK \| Peroxiredoxin-1 OS=Gallus gallus OX=9031 GN=PRDX1 PE=1 SV=1 \| \| 151 \| sp\|Q98TF6\|RL36_CHICK \| 60S ribosomal protein L36 OS=Gallus gallus OX=9031 GN=RPL36 PE=3 SV=1 \| \| 152 \| tr\|A0A1D5NXS9\|A0A1D5NXS9_CHICK \| Alpha-actinin-4 OS=Gallus gallus OX=9031 GN=ACTN4 PE=3 SV=2 \| \| 153 \| tr\|A0A1D5PII6\|A0A1D5PII6_CHICK \| RuvB-like helicase OS=Gallus gallus OX=9031 GN=LOC107051177 PE=3 SV=2 \| \| 154 \| tr\|A0A1D5P2Y8\|A0A1D5P2Y8_CHICK \| Eukaryotic initiation factor 4A-II OS=Gallus gallus OX=9031 GN=EIF4A2 PE=3 SV=1 \| \| 155 \| tr\|A0A3Q3A7C4\|A0A3Q3A7C4_CHICK \| Uncharacterized protein OS=Gallus gallus OX=9031 GN=LOC107049717 PE=3 SV=1 \| \| 156 \| sp\|P0C1H3\|H2B1_CHICK \| Histone H2B 1/2/3/4/6 OS=Gallus gallus OX=9031 GN=H2B-VI PE=1 SV=2 \| \| 158 \| tr\|E1C2L5\|E1C2L5_CHICK \| RRM domain-containing protein OS=Gallus gallus OX=9031 GN=ALYREF PE=4 SV=3 \| \| 159 \| tr\|A0A1D5PE74\|A0A1D5PE74_CHICK \| Fibrillar collagen NC1 domain-containing protein OS=Gallus gallus OX=9031 GN=COL5A1 PE=4 SV=2 \| \| 161 \| sp\|P14315\|CAPZB_CHICK \| F-actin-capping protein subunit beta isoforms 1 and 2 OS=Gallus gallus OX=9031 GN=CAPZB PE=1 SV=3 \| \| 162 \| sp\|P30352\|SRSF2_CHICK \| Serine/arginine-rich splicing factor 2 OS=Gallus gallus OX=9031 GN=SRSF2 PE=2 SV=1 \| \| 163 \| sp\|P33150\|CAD13_CHICK \| Cadherin-13 OS=Gallus gallus OX=9031 GN=CDH13 PE=1 SV=1 \| \| 164 \| tr\|A0A3Q2TYK7\|A0A3Q2TYK7_CHICK \| Actin-related protein 2 OS=Gallus gallus OX=9031 GN=ACTR2 PE=3 SV=1 \| \| 165 \| tr\|E1BS06\|E1BS06_CHICK \| Ribosomal_L23eN domain-containing protein OS=Gallus gallus OX=9031 GN=RPL23A PE=3 SV=2 \| \| 166 \| tr\|A0A3Q2U4N9\|A0A3Q2U4N9_CHICK \| Uncharacterized protein OS=Gallus gallus OX=9031 GN=LOC425607 PE=3 SV=1 \| \| 167 \| tr\|A0A452J7Z6\|A0A452J7Z6_CHICK \| Calponin OS=Gallus gallus OX=9031 GN=CNN1 PE=3 SV=1 \| \| 168 \| tr\|F1NJF0\|F1NJF0_CHICK \| Complement component 1 Q subcomponent-binding protein, mitochondrial OS=Gallus gallus OX=9031 GN=C1QBP PE=3 SV=3 \| \| 169 \| sp\|P67883\|RL30_CHICK \| 60S ribosomal protein L30 OS=Gallus gallus OX=9031 GN=RPL30 PE=1 SV=2 \| \| 170 \| tr\|F6SU35\|F6SU35_CHICK \| Ribosomal protein OS=Gallus gallus OX=9031 GN=RPL10A PE=3 SV=1 \| \| 171 \| tr\|A0A1D5P6Q5\|A0A1D5P6Q5_CHICK \| Fatty acid synthase OS=Gallus gallus OX=9031 GN=FASN PE=4 SV=1 \| \| 172 \| tr\|A0A1D5NVZ1\|A0A1D5NVZ1_CHICK \| Uncharacterized protein OS=Gallus gallus OX=9031 GN=FAM120C PE=3 SV=2 \| \| 173 \| sp\|Q5ZKD7\|MOV10_CHICK \| Putative helicase MOV-10 OS=Gallus gallus OX=9031 GN=MOV10 PE=2 SV=1 \| \| 174 \| tr\|A0A1D5P017\|A0A1D5P017_CHICK \| Myosin_tail_1 domain-containing protein OS=Gallus gallus OX=9031 GN=CCDC102A PE=4 SV=3 \| \| 175 \| tr\|A0A3Q2U427\|A0A3Q2U427_CHICK \| SMC hinge domain-containing protein OS=Gallus gallus OX=9031 GN=SMCHD1 PE=4 SV=1 \| \| 177 \| tr\|A0A1L1RP66\|A0A1L1RP66_CHICK \| AAA domain-containing protein OS=Gallus gallus OX=9031 GN=PSMC6 PE=3 SV=1 \| \| 178 \| tr\|A0A3Q2UHM4\|A0A3Q2UHM4_CHICK \| Dynamin-1-like protein OS=Gallus gallus OX=9031 GN=DNM1L PE=3 SV=1 \| \| 179 \| tr\|F1NF19\|F1NF19_CHICK \| Synaptic functional regulator FMR1 OS=Gallus gallus OX=9031 GN=FMR1 PE=3 SV=4 \| \| 180 \| tr\|A0A3Q3B093\|A0A3Q3B093_CHICK \| Uncharacterized protein OS=Gallus gallus OX=9031 GN=TNNI1 PE=4 SV=1 \| \| 181 \| tr\|A0A1D5P4V9\|A0A1D5P4V9_CHICK \| 40S ribosomal protein S24 OS=Gallus gallus OX=9031 GN=RPS24 PE=3 SV=2 \| \| 182 \| tr\|F1P0Z7\|F1P0Z7_CHICK \| Uncharacterized protein OS=Gallus gallus OX=9031 GN=ARL8B PE=3 SV=2 \| \| 183 \| tr\|R4GGJ0\|R4GGJ0_CHICK \| Uncharacterized protein OS=Gallus gallus OX=9031 GN=RPS16 PE=3 SV=1 \| \| 184 \| tr\|A0A1L1RLH7\|A0A1L1RLH7_CHICK \| 26S proteasome regulatory subunit 4 OS=Gallus gallus OX=9031 GN=PSMC1 PE=3 SV=2 \| \| 185 \| tr\|F1NMF6\|F1NMF6_CHICK \| Procollagen-lysine 5-dioxygenase OS=Gallus gallus OX=9031 GN=PLOD1 PE=4 SV=1 \| \| 186 \| tr\|A0A1D5PY15\|A0A1D5PY15_CHICK \| Coronin OS=Gallus gallus OX=9031 GN=LOC107056441 PE=3 SV=2 \| \| 187 \| tr\|E1BUD3\|E1BUD3_CHICK \| Troponin I, fast skeletal muscle OS=Gallus gallus OX=9031 GN=TNNI2 PE=4 SV=4 \| \| 188 \| sp\|Q800K9\|SURF4_CHICK \| Surfeit locus protein 4 OS=Gallus gallus OX=9031 GN=SURF4 PE=2 SV=1 \| \| 189 \| tr\|A0A1D5PM74\|A0A1D5PM74_CHICK \| Non-specific serine/threonine protein kinase OS=Gallus gallus OX=9031 GN=CDC42BPB PE=3 SV=1 \| \| 190 \| tr\|Q9YHD2\|Q9YHD2_CHICK \| Nuclear calmodulin-binding protein (Fragment) OS=Gallus gallus OX=9031 GN=URP PE=2 SV=1 \| \| 191 \| sp\|P79781\|RS27A_CHICK \| Ubiquitin-40S ribosomal protein S27a OS=Gallus gallus OX=9031 GN=RPS27A PE=1 SV=3 \| \| 192 \| tr\|F1NUY5\|F1NUY5_CHICK \| UBX domain-containing protein OS=Gallus gallus OX=9031 GN=FAF2 PE=4 SV=2 \| \| 193 \| tr\|A0A1D5NUC9\|A0A1D5NUC9_CHICK \| 60S ribosomal protein L21 OS=Gallus gallus OX=9031 GN=RPL21 PE=3 SV=2 \| \| 194 \| tr\|Q90838\|Q90838_CHICK \| Leucine zipper protein OS=Gallus gallus OX=9031 GN=PTRF PE=2 SV=1 \| \| 195 \| tr\|E1BZS2\|E1BZS2_CHICK \| Uncharacterized protein OS=Gallus gallus OX=9031 GN=NAP1L1 PE=3 SV=1 \| \| 196 \| sp\|P02588\|TNNC2_CHICK \| Troponin C, skeletal muscle OS=Gallus gallus OX=9031 GN=TNNC2 PE=1 SV=3 \| \| 197 \| tr\|A0A1D5NZ30\|A0A1D5NZ30_CHICK \| Nucleolin OS=Gallus gallus OX=9031 GN=NCL PE=4 SV=1 \| \| 198 \| tr\|A0A1D5PIX9\|A0A1D5PIX9_CHICK \| Eukaryotic translation initiation factor 3 subunit E OS=Gallus gallus OX=9031 GN=EIF3E PE=3 SV=2 \| \| 199 \| tr\|F1NBD0\|F1NBD0_CHICK \| PHB domain-containing protein OS=Gallus gallus OX=9031 GN=ERLIN2 PE=3 SV=2 \| \| 200 \| tr\|A0A452J7V2\|A0A452J7V2_CHICK \| Ras-related protein Rab-2A OS=Gallus gallus OX=9031 GN=RAB2A PE=4 SV=1 \| \| 201 \| tr\|F1N9S7\|F1N9S7_CHICK \| Annexin OS=Gallus gallus OX=9031 GN=ANXA1 PE=3 SV=1 \| \| 202 \| tr\|A0A1D5P3N9\|A0A1D5P3N9_CHICK \| HECT domain-containing protein OS=Gallus gallus OX=9031 GN=LOC422513 PE=4 SV=1 \| \| 203 \| tr\|A0M8U0\|A0M8U0_CHICK \| F-actin-capping protein subunit alpha OS=Gallus gallus OX=9031 GN=CAPZA2 PE=3 SV=1 \| \| 204 \| tr\|A0A0E4AX31\|A0A0E4AX31_CHICK \| Enigma 90kDa large protein OS=Gallus gallus OX=9031 GN=PDLIM7 PE=2 SV=1 \| \| 206 \| tr\|A0A1D5PAI1\|A0A1D5PAI1_CHICK \| 39S ribosomal protein L34, mitochondrial OS=Gallus gallus OX=9031 GN=MRPL34 PE=3 SV=1 \| \| 207 \| tr\|A0A1D5P2W2\|A0A1D5P2W2_CHICK \| Uncharacterized protein OS=Gallus gallus OX=9031 GN=ESYT2 PE=3 SV=1 \| \| 208 \| tr\|A0A1D5NXL7\|A0A1D5NXL7_CHICK \| PCI domain-containing protein OS=Gallus gallus OX=9031 GN=PSMD3 PE=3 SV=1 \| \| 209 \| tr\|Q5ZJK3\|Q5ZJK3_CHICK \| Signal transducer and activator of transcription OS=Gallus gallus OX=9031 GN=STAT1 PE=2 SV=1 \| \| 210 \| tr\|A0A1D5P2Q1\|A0A1D5P2Q1_CHICK \| SAC domain-containing protein OS=Gallus gallus OX=9031 GN=SACM1L PE=4 SV=2 \| \| 211 \| tr\|A0A3Q2UHE4\|A0A3Q2UHE4_CHICK \| SH3 domain-containing protein OS=Gallus gallus OX=9031 GN=CTTN PE=4 SV=1 \| \| 212 \| tr\|A0A3Q2U6I8\|A0A3Q2U6I8_CHICK \| Uncharacterized protein OS=Gallus gallus OX=9031 GN=ARF3 PE=3 SV=1 \| \| 214 \| tr\|A0A2R3TVM2\|A0A2R3TVM2_CHICK \| Interferon-induced protein 35 OS=Gallus gallus OX=9031 GN=IFI35 PE=2 SV=1 \| \| 215 \| tr\|Q5ZMB8\|Q5ZMB8_CHICK \| 26S proteasome regulatory subunit 7 OS=Gallus gallus OX=9031 GN=PSMC2 PE=2 SV=1 \| \| 216 \| tr\|E1BV44\|E1BV44_CHICK \| Exportin-2 OS=Gallus gallus OX=9031 GN=CSE1L PE=3 SV=2 \| \| 217 \| tr\|A0A1D5PPG6\|A0A1D5PPG6_CHICK \| Uncharacterized protein OS=Gallus gallus OX=9031 GN=EIF2B2 PE=3 SV=2 \| \| 218 \| tr\|A0A3Q3ANM5\|A0A3Q3ANM5_CHICK \| Actin-related protein 2/3 complex subunit 4 OS=Gallus gallus OX=9031 GN=ARPC4 PE=3 SV=1 \| \| 219 \| tr\|F1NK96\|F1NK96_CHICK \| Protein disulfide-isomerase A6 OS=Gallus gallus OX=9031 GN=PDIA6 PE=3 SV=1 \| \| 220 \| sp\|Q9DF58\|ILK_CHICK \| Integrin-linked protein kinase OS=Gallus gallus OX=9031 GN=ILK PE=1 SV=1 \| \| 221 \| sp\|P23614\|BASP1_CHICK \| Brain acid soluble protein 1 homolog OS=Gallus gallus OX=9031 GN=BASP1 PE=2 SV=4 \| \| 222 \| sp\|Q08200\|RL10_CHICK \| 60S ribosomal protein L10 (Fragment) OS=Gallus gallus OX=9031 GN=RPL10 PE=2 SV=1 \| \| 223 \| tr\|A0A1D5P4R2\|A0A1D5P4R2_CHICK \| Procollagen-proline 4-dioxygenase OS=Gallus gallus OX=9031 GN=P4HA1 PE=3 SV=2 \| \| 224 \| sp\|Q5ZJ54\|TCPZ_CHICK \| T-complex protein 1 subunit zeta OS=Gallus gallus OX=9031 GN=CCT6 PE=1 SV=3 \| \| 225 \| tr\|A0A1D5PS29\|A0A1D5PS29_CHICK \| Elongation factor 2 OS=Gallus gallus OX=9031 GN=EEF2 PE=3 SV=1 \| \| 226 \| tr\|A0A1D5NZC1\|A0A1D5NZC1_CHICK \| Uncharacterized protein OS=Gallus gallus OX=9031 GN=EIF4G3 PE=3 SV=2 \| \| 229 \| tr\|Q5ZM58\|Q5ZM58_CHICK \| Uncharacterized protein OS=Gallus gallus OX=9031 GN=ACTR1A PE=2 SV=1 \| \| 230 \| tr\|F1NS24\|F1NS24_CHICK \| zinc_ribbon_16 domain-containing protein OS=Gallus gallus OX=9031 GN=MIOS PE=3 SV=4 \| \| 231 \| tr\|A0A1D5PL38\|A0A1D5PL38_CHICK \| Delta-1-pyrroline-5-carboxylate synthase OS=Gallus gallus OX=9031 GN=ALDH18A1 PE=3 SV=2 \| \| 232 \| tr\|A0A3Q3B2M9\|A0A3Q3B2M9_CHICK \| TOG domain-containing protein OS=Gallus gallus OX=9031 GN=GCN1 PE=3 SV=1 \| \| 233 \| tr\|Q5ZM66\|Q5ZM66_CHICK \| 40S ribosomal protein S26 OS=Gallus gallus OX=9031 GN=RPS26 PE=2 SV=1 \| \| 234 \| tr\|A0A1D5NUY5\|A0A1D5NUY5_CHICK \| Spectrin beta chain OS=Gallus gallus OX=9031 GN=SPTBN1 PE=3 SV=2 \| \| 235 \| tr\|F1NHR3\|F1NHR3_CHICK \| VLIG-type G domain-containing protein OS=Gallus gallus OX=9031 GN=GVINP1 PE=3 SV=3 \| \| 236 \| tr\|A0A1D5PFY6\|A0A1D5PFY6_CHICK \| HTH La-type RNA-binding domain-containing protein OS=Gallus gallus OX=9031 GN=LARP1 PE=4 SV=2 \| \| 237 \| tr\|A0A3Q2U1C6\|A0A3Q2U1C6_CHICK \| Coronin OS=Gallus gallus OX=9031 GN=CORO2A PE=3 SV=1 \| \| 238 \| tr\|F1NW48\|F1NW48_CHICK \| Uncharacterized protein OS=Gallus gallus OX=9031 GN=ACTR3B PE=3 SV=3 \| \| 239 \| tr\|A0A3Q2TZA6\|A0A3Q2TZA6_CHICK \| AAA domain-containing protein OS=Gallus gallus OX=9031 GN=PSMC5 PE=3 SV=1 \| \| 240 \| sp\|Q5ZHZ0\|DX39B_CHICK \| Spliceosome RNA helicase DDX39B OS=Gallus gallus OX=9031 GN=DDX39B PE=2 SV=1 \| \| 241 \| tr\|A0A1D5P7R1\|A0A1D5P7R1_CHICK \| RNA transcription, translation and transport factor protein OS=Gallus gallus OX=9031 GN=C5H14orf166 PE=3 SV=1 \| \| 242 \| tr\|A0A6G9IUP1\|A0A6G9IUP1_CHICK \| Bone marrow stromal antigen 2 OS=Gallus gallus OX=9031 GN=BST-2 PE=2 SV=1 \| \| 243 \| tr\|A0A1D5NZS8\|A0A1D5NZS8_CHICK \| AAA domain-containing protein OS=Gallus gallus OX=9031 GN=ATAD3B PE=4 SV=1 \| \| 244 \| tr\|A0A1D5NXB3\|A0A1D5NXB3_CHICK \| Uncharacterized protein OS=Gallus gallus OX=9031 GN=LOC100859276 PE=4 SV=2 \| \| 247 \| tr\|A0A1D5PQ16\|A0A1D5PQ16_CHICK \| Unconventional myosin-VI OS=Gallus gallus OX=9031 GN=MYO6 PE=3 SV=2 \| \| 248 \| tr\|A0A1D5P5X1\|A0A1D5P5X1_CHICK \| Uncharacterized protein OS=Gallus gallus OX=9031 GN=RCN2 PE=4 SV=2 \| \| 249 \| tr\|A0A3Q2UIA2\|A0A3Q2UIA2_CHICK \| Uncharacterized protein OS=Gallus gallus OX=9031 GN=LLGL1 PE=3 SV=1 \| \| 250 \| tr\|A0A1D5PHC5\|A0A1D5PHC5_CHICK \| Heat shock cognate protein HSP 90-beta OS=Gallus gallus OX=9031 GN=HSP90AB1 PE=3 SV=1 \| \| 251 \| tr\|F1NI29\|F1NI29_CHICK \| Enoyl-CoA hydratase OS=Gallus gallus OX=9031 GN=HADHA PE=3 SV=3 \| \| 252 \| tr\|A0A1D5PTR7\|A0A1D5PTR7_CHICK \| Translocon-associated protein subunit alpha OS=Gallus gallus OX=9031 GN=SSR1 PE=3 SV=2 \| \| 253 \| sp\|P17153\|ANXA5_CHICK \| Annexin A5 OS=Gallus gallus OX=9031 GN=ANXA5 PE=1 SV=2 \| \| 255 \| tr\|F1NH93\|F1NH93_CHICK \| 40S ribosomal protein S20 OS=Gallus gallus OX=9031 GN=RPS20 PE=3 SV=3 \| \| 256 \| tr\|A0A3Q2U2B5\|A0A3Q2U2B5_CHICK \| Matrix metalloproteinase-9 OS=Gallus gallus OX=9031 GN=MMP9 PE=3 SV=1 \| \| 257 \| tr\|Q5ZIW8\|Q5ZIW8_CHICK \| Uncharacterized protein OS=Gallus gallus OX=9031 GN=RRAS PE=2 SV=1 \| \| 259 \| sp\|Q5ZIA5\|COPB_CHICK \| Coatomer subunit beta OS=Gallus gallus OX=9031 GN=COPB1 PE=2 SV=1 \| \| 260 \| tr\|F1NMT5\|F1NMT5_CHICK \| Ras-related protein Rab-10 OS=Gallus gallus OX=9031 GN=RAB10 PE=4 SV=3 \| \| 261 \| tr\|Q5ZL80\|Q5ZL80_CHICK \| Uncharacterized protein OS=Gallus gallus OX=9031 GN=HNRNPM PE=2 SV=1 \| \| 262 \| tr\|Q5F424\|Q5F424_CHICK \| T-complex protein 1 subunit beta OS=Gallus gallus OX=9031 GN=CCT2 PE=2 SV=1 \| \| 263 \| tr\|A0A1D5PJR5\|A0A1D5PJR5_CHICK \| Arginine--tRNA ligase, cytoplasmic OS=Gallus gallus OX=9031 GN=RARS PE=3 SV=1 \| \| 264 \| tr\|A0A1D5PA60\|A0A1D5PA60_CHICK \| Methionine--tRNA ligase, cytoplasmic OS=Gallus gallus OX=9031 GN=MARS PE=3 SV=2 \| \| 265 \| tr\|F1N8Z4\|F1N8Z4_CHICK \| RuvB-like helicase OS=Gallus gallus OX=9031 GN=RUVBL1 PE=3 SV=1 \| \| 266 \| tr\|A0A1D5PNC6\|A0A1D5PNC6_CHICK \| 40S ribosomal protein S12 OS=Gallus gallus OX=9031 GN=RPS12 PE=3 SV=2 \| \| 267 \| tr\|Q5ZL58\|Q5ZL58_CHICK \| Small nuclear ribonucleoprotein Sm D3 OS=Gallus gallus OX=9031 GN=SNRPD3 PE=2 SV=1 \| \| 268 \| tr\|A0A1D5P3S9\|A0A1D5P3S9_CHICK \| Uncharacterized protein OS=Gallus gallus OX=9031 GN=ECI1 PE=3 SV=1 \| \| 269 \| tr\|F1NB52\|F1NB52_CHICK \| Coatomer subunit gamma OS=Gallus gallus OX=9031 GN=COPG1 PE=3 SV=1 \| \| 270 \| tr\|E1BQC5\|E1BQC5_CHICK \| Poly [ADP-ribose] polymerase OS=Gallus gallus OX=9031 GN=PARP14 PE=3 SV=5 \| \| 271 \| tr\|A0A3Q2U5L4\|A0A3Q2U5L4_CHICK \| MFS domain-containing protein OS=Gallus gallus OX=9031 GN=SLC2A6 PE=4 SV=1 \| \| 272 \| tr\|A0A1D5PV06\|A0A1D5PV06_CHICK \| Protein disulfide-isomerase OS=Gallus gallus OX=9031 GN=P4HB PE=3 SV=2 \| \| 273 \| tr\|A0A1D5NTA5\|A0A1D5NTA5_CHICK \| T-complex protein 1 subunit theta OS=Gallus gallus OX=9031 GN=CCT8 PE=3 SV=2 \| \| 274 \| tr\|F1NR71\|F1NR71_CHICK \| MPN domain-containing protein OS=Gallus gallus OX=9031 GN=PSMD7 PE=3 SV=4 \| \| 275 \| tr\|A0A1D5PU96\|A0A1D5PU96_CHICK \| Ubiquitin carboxyl-terminal hydrolase OS=Gallus gallus OX=9031 GN=USP10 PE=3 SV=2 \| \| 276 \| sp\|Q8JG64\|PDIA3_CHICK \| Protein disulfide-isomerase A3 OS=Gallus gallus OX=9031 GN=PDIA3 PE=2 SV=1 \| \| 277 \| tr\|A0A1D5PN97\|A0A1D5PN97_CHICK \| Methylthioribose-1-phosphate isomerase OS=Gallus gallus OX=9031 GN=MRI1 PE=3 SV=2 \| \| 278 \| tr\|A0A1L1RNZ5\|A0A1L1RNZ5_CHICK \| AAA domain-containing protein OS=Gallus gallus OX=9031 GN=RFC5 PE=3 SV=2 \| \| 279 \| tr\|F1NQ35\|F1NQ35_CHICK \| 60S ribosomal protein L35a OS=Gallus gallus OX=9031 GN=RPL35A PE=3 SV=3 \| \| 280 \| tr\|A0A1L1RKY9\|A0A1L1RKY9_CHICK \| Caldesmon OS=Gallus gallus OX=9031 GN=CALD1 PE=4 SV=2 \| \| 281 \| tr\|E1BVT3\|E1BVT3_CHICK \| Malate dehydrogenase OS=Gallus gallus OX=9031 GN=MDH2 PE=3 SV=3 \| \| 282 \| tr\|A0A3Q2UJ04\|A0A3Q2UJ04_CHICK \| Uncharacterized protein OS=Gallus gallus OX=9031 GN=PDLIM5 PE=4 SV=1 \| \| 284 \| tr\|A0A146F010\|A0A146F010_CHICK \| Type II alpha-keratin IIB OS=Gallus gallus OX=9031 GN=KRT75 PE=2 SV=1 \| \| 285 \| tr\|A0A1D5P954\|A0A1D5P954_CHICK \| Uncharacterized protein OS=Gallus gallus OX=9031 GN=HNRNPR PE=4 SV=3 \| \| 286 \| tr\|A0A1D5P2U7\|A0A1D5P2U7_CHICK \| Uncharacterized protein OS=Gallus gallus OX=9031 GN=IQGAP2 PE=4 SV=1 \| \| 287 \| tr\|F1NN16\|F1NN16_CHICK \| 40S ribosomal protein S7 OS=Gallus gallus OX=9031 GN=RPS7 PE=3 SV=3 \| \| 288 \| tr\|A0A1D5NT41\|A0A1D5NT41_CHICK \| Uncharacterized protein OS=Gallus gallus OX=9031 GN=MYOF PE=3 SV=2 \| \| 289 \| tr\|A0A3Q2U4H1\|A0A3Q2U4H1_CHICK \| Uncharacterized protein OS=Gallus gallus OX=9031 GN=ESYT1 PE=3 SV=1 \| \| 290 \| tr\|A0A1D5PXH1\|A0A1D5PXH1_CHICK \| Uncharacterized protein OS=Gallus gallus OX=9031 GN=HNRNPA3 PE=4 SV=2 \| \| 292 \| tr\|F1NPD3\|F1NPD3_CHICK \| 60S ribosomal protein L18a OS=Gallus gallus OX=9031 GN=RPL18A PE=3 SV=4 \| \| 293 \| tr\|F1ND93\|F1ND93_CHICK \| Uncharacterized protein OS=Gallus gallus OX=9031 GN=PTCD3 PE=3 SV=4 \| \| 294 \| tr\|F1NPT9\|F1NPT9_CHICK \| NADH-cytochrome b5 reductase OS=Gallus gallus OX=9031 GN=LOC112529965 PE=3 SV=4 \| \| 295 \| tr\|A0A3Q2UDS6\|A0A3Q2UDS6_CHICK \| Uncharacterized protein OS=Gallus gallus OX=9031 GN=LOC107053353 PE=3 SV=1 \| \| 296 \| tr\|F1P1K3\|F1P1K3_CHICK \| Arp2/3 complex 34 kDa subunit OS=Gallus gallus OX=9031 GN=ARPC2 PE=3 SV=3 \| \| 297 \| tr\|A0A1L1RZS6\|A0A1L1RZS6_CHICK \| Uncharacterized protein OS=Gallus gallus OX=9031 GN=THRAP3 PE=3 SV=2 \| \| 298 \| tr\|A0A1L1RMP9\|A0A1L1RMP9_CHICK \| Uncharacterized protein OS=Gallus gallus OX=9031 GN=ARL1 PE=3 SV=2 \| \| 299 \| tr\|A0A1D5PVL4\|A0A1D5PVL4_CHICK \| MPN domain-containing protein OS=Gallus gallus OX=9031 GN=EIF3F PE=4 SV=2 \| \| 300 \| tr\|F1P3C0\|F1P3C0_CHICK \| AAA domain-containing protein OS=Gallus gallus OX=9031 GN=RFC3 PE=4 SV=1 \| \| 301 \| tr\|A0A1D5PNK3\|A0A1D5PNK3_CHICK \| Uncharacterized protein OS=Gallus gallus OX=9031 GN=SLC25A11 PE=3 SV=2 \| \| 302 \| tr\|A0A3Q2TYL2\|A0A3Q2TYL2_CHICK \| Prolyl 4-hydroxylase subunit alpha-2 OS=Gallus gallus OX=9031 GN=P4HA2 PE=4 SV=1 \| \| 303 \| tr\|F2Z4K6\|F2Z4K6_CHICK \| KOW domain-containing protein OS=Gallus gallus OX=9031 GN=RPL26L1 PE=3 SV=1 \| \| 304 \| tr\|A0A1D5PMH0\|A0A1D5PMH0_CHICK \| Uncharacterized protein OS=Gallus gallus OX=9031 GN=VPS45 PE=3 SV=2 \| \| 305 \| tr\|Q5F346\|Q5F346_CHICK \| 60S ribosomal protein L28 OS=Gallus gallus OX=9031 GN=RCJMB04_34p20 PE=2 SV=1 \| \| 306 \| tr\|A0A1D5PQC3\|A0A1D5PQC3_CHICK \| Spectrin alpha chain, non-erythrocytic 1 OS=Gallus gallus OX=9031 GN=SPTAN1 PE=3 SV=2 \| \| 307 \| tr\|A0A1L1RUT1\|A0A1L1RUT1_CHICK \| Coatomer subunit beta~ OS=Gallus gallus OX=9031 GN=COPB2 PE=3 SV=2 \| \| 308 \| tr\|A0A1D5PDK5\|A0A1D5PDK5_CHICK \| Calponin-homology (CH) domain-containing protein OS=Gallus gallus OX=9031 GN=IGSF9 PE=4 SV=3 \| \| 309 \| tr\|A0A3Q2U2I5\|A0A3Q2U2I5_CHICK \| Rib_recp_KP_reg domain-containing protein OS=Gallus gallus OX=9031 GN=RRBP1 PE=4 SV=1 \| \| 310 \| sp\|Q9PV94\|RSMB_CHICK \| Small nuclear ribonucleoprotein-associated protein B~ OS=Gallus gallus OX=9031 GN=SNRPB PE=2 SV=1 \| \| 311 \| tr\|A0A1D5PV80\|A0A1D5PV80_CHICK \| Vang-like protein OS=Gallus gallus OX=9031 GN=VANGL2 PE=3 SV=2 \| \| 312 \| tr\|A0A1D5PM72\|A0A1D5PM72_CHICK \| Uncharacterized protein OS=Gallus gallus OX=9031 GN=HSD17B12 PE=3 SV=2 \| \| 313 \| tr\|A0A3Q2U225\|A0A3Q2U225_CHICK \| DNA topoisomerase 2 OS=Gallus gallus OX=9031 GN=TOP2B PE=3 SV=1 \| \| 314 \| tr\|F1NV37\|F1NV37_CHICK \| Uncharacterized protein OS=Gallus gallus OX=9031 GN=NT5C1B PE=4 SV=2 \| \| 315 \| tr\|A0A1D5NZU0\|A0A1D5NZU0_CHICK \| Uncharacterized protein OS=Gallus gallus OX=9031 GN=SEH1L PE=3 SV=1 \| \| 316 \| tr\|Q6LDD0\|Q6LDD0_CHICK \| Vinculin (Fragment) OS=Gallus gallus OX=9031 GN=vinculin PE=3 SV=1 \| \| 317 \| tr\|A0A1D5PMQ5\|A0A1D5PMQ5_CHICK \| IF rod domain-containing protein OS=Gallus gallus OX=9031 GN=LOC420039 PE=3 SV=3 \| \| 318 \| tr\|A0A1L1RMT6\|A0A1L1RMT6_CHICK \| Uncharacterized protein OS=Gallus gallus OX=9031 GN=GNAI3 PE=4 SV=2 \| \| 319 \| tr\|A0A1D5PIN7\|A0A1D5PIN7_CHICK \| RNA helicase OS=Gallus gallus OX=9031 GN=DHX30 PE=3 SV=1 \| \| 320 \| tr\|F1NVG0\|F1NVG0_CHICK \| Ig-like domain-containing protein OS=Gallus gallus OX=9031 GN=ISLR PE=4 SV=4 \| \| 323 \| tr\|A0A1D5PRI4\|A0A1D5PRI4_CHICK \| MMS19 nucleotide excision repair protein OS=Gallus gallus OX=9031 GN=MMS19 PE=3 SV=1 \| \| 324 \| tr\|A0A1D5P407\|A0A1D5P407_CHICK \| Uncharacterized protein OS=Gallus gallus OX=9031 GN=SRSF5A PE=3 SV=1 \| \| 326 \| tr\|F1NPF0\|F1NPF0_CHICK \| Protein-synthesizing GTPase OS=Gallus gallus OX=9031 GN=EIF2S3 PE=4 SV=1 \| \| 327 \| tr\|A0A3Q2U766\|A0A3Q2U766_CHICK \| Uncharacterized protein OS=Gallus gallus OX=9031 GN=PSMD11 PE=3 SV=1 \| \| 328 \| tr\|A0A3Q2TX99\|A0A3Q2TX99_CHICK \| Epithelial membrane protein 2 OS=Gallus gallus OX=9031 GN=EMP2 PE=3 SV=1 \| \| 329 \| tr\|F1NA76\|F1NA76_CHICK \| GOLD domain-containing protein OS=Gallus gallus OX=9031 GN=TMED10 PE=3 SV=1 \| \| 330 \| tr\|F1P1L5\|F1P1L5_CHICK \| Uncharacterized protein OS=Gallus gallus OX=9031 GN=TNFAIP2 PE=3 SV=2 \| \| 331 \| tr\|A0A1D5P893\|A0A1D5P893_CHICK \| Uncharacterized protein OS=Gallus gallus OX=9031 GN=LOC426023 PE=4 SV=2 \| \| 332 \| tr\|R4GJN4\|R4GJN4_CHICK \| Uncharacterized protein OS=Gallus gallus OX=9031 GN=GBE PE=4 SV=2 \| \| 333 \| tr\|R4GM10\|R4GM10_CHICK \| Fructose-bisphosphate aldolase OS=Gallus gallus OX=9031 GN=ALDOC PE=3 SV=1 \| \| 334 \| sp\|E1BTG2\|LMOD2_CHICK \| Leiomodin-2 OS=Gallus gallus OX=9031 GN=LMOD2 PE=1 SV=2 \| \| 335 \| tr\|F1NWX7\|F1NWX7_CHICK \| Protein transport protein Sec61 subunit beta OS=Gallus gallus OX=9031 GN=SEC61B PE=3 SV=2 \| \| 336 \| tr\|A0A3Q2U489\|A0A3Q2U489_CHICK \| Sas10 domain-containing protein OS=Gallus gallus OX=9031 GN=UTP3 PE=3 SV=1 \| \| 337 \| tr\|A0A1L1RXY9\|A0A1L1RXY9_CHICK \| 116 kDa U5 small nuclear ribonucleoprotein component OS=Gallus gallus OX=9031 GN=EFTUD2 PE=3 SV=2 \| \| 338 \| tr\|F1NUI2\|F1NUI2_CHICK \| Uncharacterized protein OS=Gallus gallus OX=9031 GN=XIRP1 PE=3 SV=2 \| \| 339 \| tr\|F1NBD7\|F1NBD7_CHICK \| Cyclin-dependent kinase 1 OS=Gallus gallus OX=9031 GN=CDK1 PE=3 SV=1 \| \| 340 \| tr\|F1NQW8\|F1NQW8_CHICK \| Splicing factor, arginine/serine-rich 1 OS=Gallus gallus OX=9031 GN=SRSF1 PE=3 SV=2 \| \| 341 \| tr\|A0A3Q2UIM7\|A0A3Q2UIM7_CHICK \| Alpha-mannosidase OS=Gallus gallus OX=9031 GN=MAN2A1 PE=3 SV=1 \| \| 343 \| sp\|Q5ZLN1\|PGAM1_CHICK \| Phosphoglycerate mutase 1 OS=Gallus gallus OX=9031 GN=PGAM1 PE=1 SV=3 \| \| 344 \| tr\|F1P0J8\|F1P0J8_CHICK \| Uncharacterized protein OS=Gallus gallus OX=9031 GN=THBS1 PE=3 SV=4 \| \| 345 \| tr\|A0A3S5ZPB7\|A0A3S5ZPB7_CHICK \| Phosphate carrier protein, mitochondrial OS=Gallus gallus OX=9031 GN=SLC25A3 PE=3 SV=1 \| \| 346 \| tr\|F1NQG5\|F1NQG5_CHICK \| Ribosomal protein L15 OS=Gallus gallus OX=9031 GN=RPL15 PE=3 SV=2 \| \| 347 \| tr\|A0A0K1YW01\|A0A0K1YW01_CHICK \| Stimulator of interferon genes protein OS=Gallus gallus OX=9031 GN=STING PE=2 SV=1 \| \| 348 \| tr\|A0A1D5PF37\|A0A1D5PF37_CHICK \| Uncharacterized protein OS=Gallus gallus OX=9031 GN=MRPL55 PE=4 SV=3 \| \| 349 \| tr\|A0A1D5P888\|A0A1D5P888_CHICK \| Serine/threonine-protein phosphatase OS=Gallus gallus OX=9031 GN=PPP1CA PE=3 SV=2 \| \| 350 \| tr\|A0A1D5PCD1\|A0A1D5PCD1_CHICK \| Flotillin OS=Gallus gallus OX=9031 GN=FLOT2 PE=3 SV=2 \| \| 351 \| tr\|A0A3Q2TZV9\|A0A3Q2TZV9_CHICK \| Calponin-homology (CH) domain-containing protein OS=Gallus gallus OX=9031 GN=LRCH3 PE=4 SV=1 \| \| 352 \| sp\|P13731\|SERPH_CHICK \| Serpin H1 OS=Gallus gallus OX=9031 GN=SERPINH1 PE=1 SV=2 \| \| 353 \| tr\|A0A1L1RX65\|A0A1L1RX65_CHICK \| Isocitrate dehydrogenase [NAD] subunit, mitochondrial OS=Gallus gallus OX=9031 GN=IDH3A PE=3 SV=2 \| \| 354 \| tr\|R4GJP9\|R4GJP9_CHICK \| Histone H2A OS=Gallus gallus OX=9031 GN=LOC101751709 PE=3 SV=2 \| \| 355 \| tr\|A0A1D5PY67\|A0A1D5PY67_CHICK \| Annexin OS=Gallus gallus OX=9031 GN=ANXA6 PE=3 SV=2 \| \| 356 \| tr\|A0A1D5NZ06\|A0A1D5NZ06_CHICK \| 40S ribosomal protein S27 OS=Gallus gallus OX=9031 GN=RPS27 PE=3 SV=1 \| \| 357 \| tr\|A0A1L1S0X5\|A0A1L1S0X5_CHICK \| 60S ribosomal protein L34 OS=Gallus gallus OX=9031 GN=RPL34 PE=3 SV=2 \| \| 358 \| tr\|A0A1D5P1P9\|A0A1D5P1P9_CHICK \| Ribonucloprotein OS=Gallus gallus OX=9031 GN=NHP2 PE=3 SV=2 \| \| 359 \| tr\|E1BW27\|E1BW27_CHICK \| Uncharacterized protein OS=Gallus gallus OX=9031 GN=TMEM33 PE=3 SV=1 \| \| 360 \| tr\|F1NV49\|F1NV49_CHICK \| ATP-dependent RNA helicase DDX1 OS=Gallus gallus OX=9031 GN=DDX1 PE=3 SV=3 \| \| 361 \| tr\|A0A3Q2UGY6\|A0A3Q2UGY6_CHICK \| C2 domain-containing protein OS=Gallus gallus OX=9031 GN=C2CD5 PE=4 SV=1 \| \| 362 \| tr\|A0A1L1RWS6\|A0A1L1RWS6_CHICK \| RNA helicase OS=Gallus gallus OX=9031 GN=DDX27 PE=3 SV=2 \| \| 363 \| tr\|F1P4X7\|F1P4X7_CHICK \| Uncharacterized protein OS=Gallus gallus OX=9031 GN=DHCR7 PE=3 SV=2 \| \| 364 \| tr\|A0A3Q2U263\|A0A3Q2U263_CHICK \| Uncharacterized protein OS=Gallus gallus OX=9031 GN=RAB11FIP1 PE=4 SV=1 \| \| 365 \| tr\|E1BZT5\|E1BZT5_CHICK \| MPN domain-containing protein OS=Gallus gallus OX=9031 GN=PRPF8 PE=4 SV=1 \| \| 366 \| tr\|A0A1D5NYB2\|A0A1D5NYB2_CHICK \| Polyadenylate-binding protein OS=Gallus gallus OX=9031 GN=PABPC1 PE=3 SV=2 \| \| 367 \| tr\|F1NSS7\|F1NSS7_CHICK \| Ribosome biogenesis protein BRX1 homolog OS=Gallus gallus OX=9031 GN=BRIX1 PE=3 SV=1 \| \| 368 \| tr\|A0A1D5NV94\|A0A1D5NV94_CHICK \| AP-2 complex subunit alpha OS=Gallus gallus OX=9031 GN=AP2A2 PE=3 SV=2 \| \| 369 \| tr\|A0A1D5NZQ6\|A0A1D5NZQ6_CHICK \| Uncharacterized protein OS=Gallus gallus OX=9031 GN=AP1B1 PE=3 SV=2 \| \| 370 \| tr\|A0A1D5NTA0\|A0A1D5NTA0_CHICK \| Ubiquitin thioesterase OS=Gallus gallus OX=9031 GN=OTUB1 PE=3 SV=1 \| \| 371 \| tr\|A0A3Q3AZJ6\|A0A3Q3AZJ6_CHICK \| RRM domain-containing protein OS=Gallus gallus OX=9031 GN=RBM3 PE=4 SV=1 \| \| 372 \| tr\|H9CX01\|H9CX01_CHICK \| Transforming growth factor beta OS=Gallus gallus OX=9031 GN=TGFB1 PE=2 SV=1 \| \| 373 \| tr\|A0A1L1RMM0\|A0A1L1RMM0_CHICK \| T-complex protein 1 subunit alpha OS=Gallus gallus OX=9031 GN=TCP1 PE=3 SV=2 \| \| 374 \| tr\|A0A452J7T4\|A0A452J7T4_CHICK \| AP-2 complex subunit mu OS=Gallus gallus OX=9031 GN=AP2M1 PE=3 SV=1 \| \| 375 \| tr\|A0A1L1RJR6\|A0A1L1RJR6_CHICK \| Uncharacterized protein OS=Gallus gallus OX=9031 GN=METTL3 PE=3 SV=2 \| \| 376 \| tr\|A0A3Q2U853\|A0A3Q2U853_CHICK \| Uncharacterized protein OS=Gallus gallus OX=9031 GN=NAP1L4 PE=3 SV=1 \| \| 377 \| tr\|A0A3Q2UHT9\|A0A3Q2UHT9_CHICK \| Uncharacterized protein OS=Gallus gallus OX=9031 GN=EHD4 PE=4 SV=1 \| \| 378 \| tr\|F1P2B1\|F1P2B1_CHICK \| Torsin OS=Gallus gallus OX=9031 GN=TOR1A PE=3 SV=1 \| \| 379 \| tr\|F1NWG6\|F1NWG6_CHICK \| Protein VAC14 homolog OS=Gallus gallus OX=9031 GN=VAC14 PE=3 SV=1 \| \| 380 \| tr\|A0A1D5P185\|A0A1D5P185_CHICK \| Coatomer subunit alpha OS=Gallus gallus OX=9031 GN=COPA PE=4 SV=1 \| \| 381 \| tr\|A0A1L1RWG9\|A0A1L1RWG9_CHICK \| IF rod domain-containing protein OS=Gallus gallus OX=9031 GN=KRT24 PE=3 SV=2 \| \| 382 \| tr\|Q5ZHM8\|Q5ZHM8_CHICK \| Uncharacterized protein OS=Gallus gallus OX=9031 GN=SDCBP PE=2 SV=1 \| \| 384 \| tr\|A0A1D5PAD1\|A0A1D5PAD1_CHICK \| Uncharacterized protein OS=Gallus gallus OX=9031 GN=MTA3 PE=4 SV=1 \| \| 385 \| tr\|A0A0D3QU53\|A0A0D3QU53_CHICK \| Cytochrome c oxidase subunit 2 OS=Gallus gallus OX=9031 GN=COX2 PE=3 SV=1 \| \| 386 \| tr\|A0A1D5P251\|A0A1D5P251_CHICK \| Uncharacterized protein OS=Gallus gallus OX=9031 GN=MAP1S PE=4 SV=2 \| \| 387 \| tr\|Q2XNL5\|Q2XNL5_CHICK \| Tumor necrosis factor-inducible protein 6 OS=Gallus gallus OX=9031 GN=TSG-6 PE=2 SV=1 \| \| 388 \| tr\|A0A1D5PDI5\|A0A1D5PDI5_CHICK \| Aldolase_II domain-containing protein OS=Gallus gallus OX=9031 GN=ADD2 PE=3 SV=2 \| \| 389 \| tr\|Q5ZJ61\|Q5ZJ61_CHICK \| Phenylalanine--tRNA ligase beta subunit OS=Gallus gallus OX=9031 GN=FARSB PE=2 SV=1 \| \| 390 \| tr\|A0A3Q2TTG0\|A0A3Q2TTG0_CHICK \| Uncharacterized protein OS=Gallus gallus OX=9031 GN=MAP2K5 PE=4 SV=1 \| \| 391 \| tr\|A0A1D5PUC0\|A0A1D5PUC0_CHICK \| H(+)-transporting two-sector ATPase OS=Gallus gallus OX=9031 GN=LOC776719 PE=3 SV=1 \| \| 392 \| tr\|A0A3Q2U943\|A0A3Q2U943_CHICK \| Uncharacterized protein OS=Gallus gallus OX=9031 GN=UBTF PE=4 SV=1 \| \| 393 \| tr\|B6ZLK1\|B6ZLK1_CHICK \| FACT complex subunit SSRP1 OS=Gallus gallus OX=9031 GN=SSRP1 PE=2 SV=1 \| \| 394 \| tr\|A0A1D5PFV9\|A0A1D5PFV9_CHICK \| Protein kinase domain-containing protein OS=Gallus gallus OX=9031 GN=SRPK1 PE=4 SV=1 \| \| 395 \| tr\|R4GM79\|R4GM79_CHICK \| DNA topoisomerase I OS=Gallus gallus OX=9031 GN=TOP1 PE=3 SV=3 \| \| 396 \| sp\|Q0GGW5\|STK11_CHICK \| Serine/threonine-protein kinase STK11 OS=Gallus gallus OX=9031 GN=STK11 PE=2 SV=1 \| \| 397 \| tr\|D2Z1L9\|D2Z1L9_CHICK \| LIM and SH3 domain protein 1 OS=Gallus gallus OX=9031 GN=LASP1 PE=2 SV=1 \| \| 398 \| sp\|P84169\|PSD13_CHICK \| 26S proteasome non-ATPase regulatory subunit 13 OS=Gallus gallus OX=9031 GN=PSMD13 PE=1 SV=1 \| \| 399 \| tr\|F1NFB8\|F1NFB8_CHICK \| E3 ubiquitin-protein ligase OS=Gallus gallus OX=9031 GN=DTX3L PE=3 SV=2 \| \| 400 \| tr\|E1BTT8\|E1BTT8_CHICK \| L-lactate dehydrogenase OS=Gallus gallus OX=9031 GN=LDHA PE=3 SV=2 \| \| 401 \| tr\|A0A1L1RRE9\|A0A1L1RRE9_CHICK \| Uncharacterized protein OS=Gallus gallus OX=9031 GN=TNS1 PE=3 SV=1 \| \| 402 \| tr\|A0A1L1RSB5\|A0A1L1RSB5_CHICK \| Sm protein F OS=Gallus gallus OX=9031 GN=SNRPF PE=3 SV=2 \| \| 403 \| tr\|A0A1D5PXT6\|A0A1D5PXT6_CHICK \| PDZ domain-containing protein OS=Gallus gallus OX=9031 GN=PDZRN4 PE=4 SV=1 \| \| 404 \| tr\|A0A3Q2U5T4\|A0A3Q2U5T4_CHICK \| Integrin_alpha2 domain-containing protein OS=Gallus gallus OX=9031 GN=ITGA2B PE=3 SV=1 \| \| 405 \| tr\|A0A3Q2U0D5\|A0A3Q2U0D5_CHICK \| Uncharacterized protein OS=Gallus gallus OX=9031 GN=ABCF2 PE=4 SV=1 \| \| 406 \| tr\|A0A1D5P7F9\|A0A1D5P7F9_CHICK \| Uncharacterized protein OS=Gallus gallus OX=9031 GN=PPFIA2 PE=3 SV=2 \| \| 407 \| tr\|E1C1Q0\|E1C1Q0_CHICK \| LIM zinc-binding domain-containing protein OS=Gallus gallus OX=9031 GN=LYAR PE=4 SV=4 \| \| 408 \| sp\|P07583\|LEG4_CHICK \| Beta-galactoside-binding lectin OS=Gallus gallus OX=9031 GN=CG-1B PE=1 SV=2 \| \| 409 \| tr\|A0A1D5NT61\|A0A1D5NT61_CHICK \| Glutamate dehydrogenase (NAD(P)(+)) OS=Gallus gallus OX=9031 GN=GLUD2 PE=3 SV=1 \| \| 410 \| tr\|A0A3Q2UPL0\|A0A3Q2UPL0_CHICK \| Meiosis-specific nuclear structural protein 1 OS=Gallus gallus OX=9031 GN=MNS1 PE=3 SV=1 \| \| 411 \| tr\|F1NU56\|F1NU56_CHICK \| 40S ribosomal protein S25 OS=Gallus gallus OX=9031 GN=RPS25 PE=3 SV=2 \| \| 412 \| tr\|A0A0A0MQ56\|A0A0A0MQ56_CHICK \| EF-hand domain-containing family member C2 OS=Gallus gallus OX=9031 GN=EFHC2 PE=4 SV=1 \| \| 413 \| tr\|A0A1L1RUB9\|A0A1L1RUB9_CHICK \| AAA domain-containing protein OS=Gallus gallus OX=9031 GN=RFC4 PE=3 SV=2 \| \| 414 \| tr\|R4GH52\|R4GH52_CHICK \| Uncharacterized protein OS=Gallus gallus OX=9031 GN=P3H4 PE=4 SV=2 \| \| 415 \| sp\|Q98TF5\|RL39_CHICK \| 60S ribosomal protein L39 OS=Gallus gallus OX=9031 GN=RPL39 PE=3 SV=1 \| \| 416 \| tr\|Q5EFL0\|Q5EFL0_CHICK \| Methyl-CpG-binding domain protein 2 OS=Gallus gallus OX=9031 GN=MBD2 PE=1 SV=1 \| \| 417 \| tr\|A0A1L1RKT0\|A0A1L1RKT0_CHICK \| Glycylpeptide N-tetradecanoyltransferase OS=Gallus gallus OX=9031 GN=NMT1 PE=3 SV=1 \| \| 418 \| sp\|P09324\|YES_CHICK \| Tyrosine-protein kinase Yes OS=Gallus gallus OX=9031 GN=YES1 PE=1 SV=3 \| \| 419 \| tr\|A0A1D5NYA0\|A0A1D5NYA0_CHICK \| NIPSNAP domain-containing protein OS=Gallus gallus OX=9031 GN=NIPSNAP1 PE=3 SV=2 \| \| 420 \| tr\|Q98TH5\|Q98TH5_CHICK \| 40S ribosomal protein S11 OS=Gallus gallus OX=9031 GN=cRPS11 PE=3 SV=1 \| \| 421 \| sp\|Q5ZMH1\|SEPT2_CHICK \| Septin-2 OS=Gallus gallus OX=9031 GN=SEPTIN2 PE=2 SV=1 \| \| 422 \| tr\|A0A1D5PU80\|A0A1D5PU80_CHICK \| Sodium/potassium-transporting ATPase subunit alpha OS=Gallus gallus OX=9031 GN=ATP1A1 PE=3 SV=1 \| \| 423 \| tr\|Q5ZM18\|Q5ZM18_CHICK \| Nucleolar GTP-binding protein 1 OS=Gallus gallus OX=9031 GN=GTPBP4 PE=2 SV=1 \| \| 424 \| tr\|A0A1D5PTP3\|A0A1D5PTP3_CHICK \| Aspartate--tRNA ligase, cytoplasmic OS=Gallus gallus OX=9031 GN=DARS PE=3 SV=1 \| \| 425 \| tr\|E1BYH2\|E1BYH2_CHICK \| Protein kinase domain-containing protein OS=Gallus gallus OX=9031 GN=TAOK1 PE=4 SV=1 \| \| 426 \| sp\|Q9PSX7\|RHOC_CHICK \| Rho-related GTP-binding protein RhoC OS=Gallus gallus OX=9031 GN=RHOC PE=1 SV=1 \| \| 427 \| tr\|Q5ZK18\|Q5ZK18_CHICK \| 3HCDH_N domain-containing protein OS=Gallus gallus OX=9031 GN=RCJMB04_13l16 PE=2 SV=1 \| \| 428 \| sp\|Q5ZIE6\|ULA1_CHICK \| NEDD8-activating enzyme E1 regulatory subunit OS=Gallus gallus OX=9031 GN=NAE1 PE=2 SV=1 \| \| 429 \| tr\|A0A1D5PA67\|A0A1D5PA67_CHICK \| Acylamino-acid-releasing enzyme OS=Gallus gallus OX=9031 GN=LOC770794 PE=3 SV=1 \| \| 432 \| tr\|A0A1D5PSE5\|A0A1D5PSE5_CHICK \| ATP-citrate synthase OS=Gallus gallus OX=9031 GN=ACLY PE=3 SV=1 \| \| 433 \| tr\|A0A1D5PBA6\|A0A1D5PBA6_CHICK \| Uncharacterized protein OS=Gallus gallus OX=9031 GN=DCTN4 PE=4 SV=2 \| \| 435 \| tr\|E1C3E1\|E1C3E1_CHICK \| N-myc proto-oncogene protein OS=Gallus gallus OX=9031 GN=MYCN PE=4 SV=3 \| |
| --- | --- | --- | --- | --- | --- | --- | --- | --- | --- | --- | --- | --- | --- | --- | --- | --- | --- | --- | --- | --- | --- | --- | --- | --- | --- | --- | --- | --- | --- | --- | --- | --- | --- | --- | --- | --- | --- | --- | --- | --- | --- | --- | --- | --- | --- | --- | --- | --- | --- | --- | --- | --- | --- | --- | --- | --- | --- | --- | --- | --- | --- | --- | --- | --- | --- | --- | --- | --- | --- | --- | --- | --- | --- | --- | --- | --- | --- | --- | --- | --- | --- | --- | --- | --- | --- | --- | --- | --- | --- | --- | --- | --- | --- | --- | --- | --- | --- | --- | --- | --- | --- | --- | --- | --- | --- | --- | --- | --- | --- | --- | --- | --- | --- | --- | --- | --- | --- | --- | --- | --- | --- | --- | --- | --- | --- | --- | --- | --- | --- | --- | --- | --- | --- | --- | --- | --- | --- | --- | --- | --- | --- | --- | --- | --- | --- | --- | --- | --- | --- | --- | --- | --- | --- | --- | --- | --- | --- | --- | --- | --- | --- | --- | --- | --- | --- | --- | --- | --- | --- | --- | --- | --- | --- | --- | --- | --- | --- | --- | --- | --- | --- | --- | --- | --- | --- | --- | --- | --- | --- | --- | --- | --- | --- | --- | --- | --- | --- | --- | --- | --- | --- | --- | --- | --- | --- | --- | --- | --- | --- | --- | --- | --- | --- | --- | --- | --- | --- | --- | --- | --- | --- | --- | --- | --- | --- | --- | --- | --- | --- | --- | --- | --- | --- | --- | --- | --- | --- | --- | --- | --- | --- | --- | --- | --- | --- | --- | --- | --- | --- | --- | --- | --- | --- | --- | --- | --- | --- | --- | --- | --- | --- | --- | --- | --- | --- | --- | --- | --- | --- | --- | --- | --- | --- | --- | --- | --- | --- | --- | --- | --- | --- | --- | --- | --- | --- | --- | --- | --- | --- | --- | --- | --- | --- | --- | --- | --- | --- | --- | --- | --- | --- | --- | --- | --- | --- | --- | --- | --- | --- | --- | --- | --- | --- | --- | --- | --- | --- | --- | --- | --- | --- | --- | --- | --- | --- | --- | --- | --- | --- | --- | --- | --- | --- | --- | --- | --- | --- | --- | --- | --- | --- | --- | --- | --- | --- | --- | --- | --- | --- | --- | --- | --- | --- | --- | --- | --- | --- | --- | --- | --- | --- | --- | --- | --- | --- | --- | --- | --- | --- | --- | --- | --- | --- | --- | --- | --- | --- | --- | --- | --- | --- | --- | --- | --- | --- | --- | --- | --- | --- | --- | --- | --- | --- | --- | --- | --- | --- | --- | --- | --- | --- | --- | --- | --- | --- | --- | --- | --- | --- | --- | --- | --- | --- | --- | --- | --- | --- | --- | --- | --- | --- | --- | --- | --- | --- | --- | --- | --- | --- | --- | --- | --- | --- | --- | --- | --- | --- | --- | --- | --- | --- | --- | --- | --- | --- | --- | --- | --- | --- | --- | --- | --- | --- | --- | --- | --- | --- | --- | --- | --- | --- | --- | --- | --- | --- | --- | --- | --- | --- | --- | --- | --- | --- | --- | --- | --- | --- | --- | --- | --- | --- | --- | --- | --- | --- | --- | --- | --- | --- | --- | --- | --- | --- | --- | --- | --- | --- | --- | --- | --- | --- | --- | --- | --- | --- | --- | --- | --- | --- | --- | --- | --- | --- | --- | --- | --- | --- | --- | --- | --- | --- | --- | --- | --- | --- | --- | --- | --- | --- | --- | --- | --- | --- | --- | --- | --- | --- | --- | --- | --- | --- | --- | --- | --- | --- | --- | --- | --- | --- | --- | --- | --- | --- | --- | --- | --- | --- | --- | --- | --- | --- | --- | --- | --- | --- | --- | --- | --- | --- | --- | --- | --- | --- | --- | --- | --- | --- | --- | --- | --- | --- | --- | --- | --- | --- | --- | --- | --- | --- | --- | --- | --- | --- | --- | --- | --- | --- | --- | --- | --- | --- | --- | --- | --- | --- | --- | --- | --- | --- | --- | --- | --- | --- | --- | --- | --- | --- | --- | --- | --- | --- | --- | --- | --- | --- | --- | --- | --- | --- | --- | --- | --- | --- | --- | --- | --- | --- | --- | --- | --- | --- | --- | --- | --- | --- | --- | --- | --- | --- | --- | --- | --- | --- | --- | --- | --- | --- | --- | --- | --- | --- | --- | --- | --- | --- | --- | --- | --- | --- | --- | --- | --- | --- | --- | --- | --- | --- | --- | --- | --- | --- | --- | --- | --- | --- | --- | --- | --- | --- | --- | --- | --- | --- | --- | --- | --- | --- | --- | --- | --- | --- | --- | --- | --- | --- | --- | --- | --- | --- | --- | --- | --- | --- | --- | --- | --- | --- | --- | --- | --- | --- | --- | --- | --- | --- | --- | --- | --- | --- | --- | --- | --- | --- | --- | --- | --- | --- | --- | --- | --- | --- | --- | --- | --- | --- | --- | --- | --- | --- | --- | --- | --- | --- | --- | --- | --- | --- | --- | --- | --- | --- | --- | --- | --- | --- | --- | --- | --- | --- | --- | --- | --- | --- | --- | --- | --- | --- | --- | --- | --- | --- | --- | --- | --- | --- | --- | --- | --- | --- | --- | --- | --- | --- | --- | --- | --- | --- | --- | --- | --- | --- | --- | --- | --- | --- | --- | --- | --- | --- | --- | --- | --- | --- | --- | --- | --- | --- | --- | --- | --- | --- | --- | --- | --- | --- | --- | --- | --- | --- | --- | --- | --- | --- | --- | --- | --- | --- | --- | --- | --- | --- | --- | --- | --- | --- | --- | --- | --- | --- | --- | --- | --- | --- | --- | --- | --- | --- | --- | --- | --- | --- | --- | --- | --- | --- | --- | --- | --- | --- | --- | --- | --- | --- | --- | --- | --- | --- | --- | --- | --- | --- | --- | --- | --- | --- | --- | --- | --- | --- | --- | --- | --- | --- | --- | --- | --- | --- | --- | --- | --- | --- | --- | --- | --- | --- | --- | --- | --- | --- | --- | --- | --- | --- | --- | --- | --- | --- | --- | --- | --- | --- | --- | --- | --- | --- | --- | --- | --- | --- | --- | --- | --- | --- | --- | --- | --- | --- | --- | --- | --- | --- | --- | --- | --- | --- | --- | --- | --- | --- | --- | --- | --- | --- | --- | --- | --- | --- | --- | --- | --- | --- | --- | --- | --- | --- | --- | --- | --- | --- | --- | --- | --- | --- | --- | --- | --- | --- | --- | --- | --- | --- | --- | --- | --- | --- | --- | --- | --- | --- | --- | --- | --- | --- | --- | --- | --- | --- | --- | --- | --- | --- | --- | --- | --- | --- | --- | --- | --- | --- | --- | --- | --- | --- | --- | --- | --- | --- | --- | --- | --- | --- | --- | --- | --- | --- | --- | --- | --- | --- | --- | --- | --- | --- | --- | --- | --- | --- | --- | --- | --- | --- | --- | --- | --- | --- | --- | --- | --- | --- | --- | --- | --- | --- | --- | --- | --- | --- | --- | --- | --- | --- | --- | --- | --- | --- | --- | --- | --- | --- | --- | --- | --- | --- | --- | --- | --- | --- | --- | --- | --- | --- | --- | --- | --- | --- | --- | --- | --- | --- | --- | --- | --- | --- | --- | --- | --- | --- | --- | --- | --- | --- | --- | --- | --- | --- | --- | --- | --- | --- | --- | --- | --- | --- | --- | --- | --- | --- | --- | --- | --- | --- | --- | --- | --- | --- | --- | --- | --- | --- | --- | --- | --- | --- | --- | --- | --- | --- | --- | --- | --- | --- | --- | --- |
